# *Lactobacillus oligofermentans* glucose, ribose and xylose transcriptomes show higher similarity between glucose and xylose catabolism-induced responses in the early exponential growth phase

**DOI:** 10.1186/s12864-016-2840-x

**Published:** 2016-08-03

**Authors:** Margarita Andreevskaya, Per Johansson, Elina Jääskeläinen, Tanja Rämö, Jarmo Ritari, Lars Paulin, Johanna Björkroth, Petri Auvinen

**Affiliations:** 1 Institute of Biotechnology, University of Helsinki, Helsinki, Finland; 2 Department of Food Hygiene and Environmental Health, University of Helsinki, Helsinki, Finland; 3 Present Address: The National Bureau of Investigation, Vantaa, Finland; 4 Present Address: Finnish Red Cross Blood Service, Helsinki, Finland

**Keywords:** *Lactobacillus oligofermentans*, Obligate heterofermentative metabolism, Carbohydrate catabolism, RNA-seq based transcriptomes, CcpA, Carbon catabolite control, Redox-sensing transcriptional repressor Rex, NAD(P)H re-oxidation

## Abstract

**Background:**

*Lactobacillus oligofermentans* has been mostly isolated from cold-stored packaged meat products in connection with their spoilage, but its precise role in meat spoilage is unknown. It belongs to the *L. vaccinostercus* group of obligate heterofermentative lactobacilli that generally ferment pentoses (e.g. xylose and ribose) more efficiently than hexoses (e.g. glucose). However, more efficient hexose utilization can be induced. The regulation mechanisms of the carbohydrate catabolism in such bacteria have been scarcely studied. To address this question, we provided the complete genome sequence of *L. oligofermentans* LMG 22743^T^ and generated time course transcriptomes during its growth on glucose, ribose and xylose.

**Results:**

The genome was manually annotated and its main functional features were examined. *L. oligofermentans* was confirmed to be able to efficiently utilize several hexoses and maltose, which is, presumably, induced by its repeated cultivation with glucose *in vitro*. Unexpectedly, in the beginning of the exponential growth phase, glucose- and xylose-induced transcriptome responses were more similar, whereas toward the end of the growth phase xylose and ribose transcriptomes became more alike. The promoter regions of genes simultaneously upregulated both on glucose and xylose in comparison with ribose (particularly, hexose and xylose utilization genes) were found to be enriched in the CcpA- binding site. Transcriptionally, no glucose-induced carbon catabolite repression was detected. The catabolism of glucose, which requires initial oxidation, led to significant overexpression of the NAD(P)H re-oxidation genes, the upstream regions of which were found to contain a motif, which was highly similar to a Rex repressor binding site.

**Conclusions:**

This paper presents the second complete genome and the first study of carbohydrate catabolism-dependent transcriptome response for a member of the *L. vaccinostercus* group. The transcriptomic changes detected in *L. oligofermentans* for growth with different carbohydrates differ significantly from those of facultative heterofermentative lactobacilli. The mechanism of CcpA regulation, putatively contributing to the observed similarities between glucose- and xylose-induced transcriptome responses and the absence of stringent carbon catabolite control, requires further studies. Finally, the cell redox balance maintenance, in terms of the NAD(P)+/NAD(P)H ratio, was predicted to be regulated by the Rex transcriptional regulator, supporting the previously made inference of Rex-regulons for members of the *Lactobacillaceae* family.

**Electronic supplementary material:**

The online version of this article (doi:10.1186/s12864-016-2840-x) contains supplementary material, which is available to authorized users.

## Background

*Lactobacillus oligofermentans* is an obligate heterofermentative lactic acid bacterium (LAB) that has been continuously detected in cold-stored modified atmosphere packaged (MAP) broiler meat products at the end of shelf life or spoilage stage [1]. Recently, it was also isolated from MAP ground beef [2] and fermented Chinese vegetables [3]. In spoiled poultry products, *L. oligofermentans* has constituted 10–18 % of spoilage LAB, though, it has never predominated the microbiota [1]. Therefore, its role in meat spoilage is still unclear.

Phylogenetically, *L. oligofermentans* is most closely related to *L. vaccinostercus, L. suebicus, L. hokkaidonensis, L. nenjiangensis* and *L. wasatchensis* (the so-called *L. vaccinostercus* group) [4, 5]*.* These bacteria represent a group of obligate heterofermentative lactobacilli, which degrade carbohydrates only through the phosphoketolase pathway, while lacking the key enzymes for glycolysis [4]. The phosphoketolase pathway is better adapted for the catabolism of pentoses, which, in comparison with hexose fermentation, yield twice as much ATP [6]. As a consequence, the growth rate and yield of these bacteria are considerably higher during the fermentation of pentoses than hexoses. On initial isolation, *L. oligofermentans* strains, including type strain LMG 22743^T^, utilized efficiently only pentoses (e.g. arabinose, ribose, xylose), while growth on hexoses was weak (e.g. glucose, N-acetylglucosamine) or negative (e.g. galactose, fructose, mannose) [1]. However, later more efficient utilization of the above-mentioned hexoses was observed for *L. oligofermentans* [7]. Pentoses are abundant in plant materials, which are the primary ecological niche for species from the *L. vaccinostercus* group (representatives have been isolated from tempoyak, apple mash, timothy grass silage, pickle, sourdough and cow dung [4, 8], while *L. wasatchensis* was isolated from cheese [5]). To our knowledge, *L. oligofermentans* is the only bacterium from this group which has repeatedly been isolated from meat sources, where concentration of free pentoses is much lower than in plant-derived materials [9], while the main fermentable carbohydrate in fresh meat is glucose [10].

In LAB, the utilization of different carbohydrates is usually regulated by the carbon catabolite repression (CCR) system [11, 12]. CCR is mediated by the catabolite control protein CcpA, which, in response to the high concentration of glycolytic intermediate fructose-1,6-bisphosphate or high levels of ATP [13], transcriptionally regulates the expression of hundreds of genes to ensure hierarchical carbohydrate utilization with glucose (most commonly) as a preferred source. However, there is very limited information about CCR and accompanying transcriptional regulation in heterofermentative bacteria lacking glycolytic intermediates and preferring pentoses to glucose. Nevertheless, all the main CCR components seem to be present in these bacteria. For *Lactobacillus brevis*, a well-studied obligate heterofermentative bacterium, the relaxed control of sugar utilization was proposed to explain the same-level utilization of glucose and other carbohydrates, including pentoses, in co-fermentation [14]. But the precise mechanism of the relaxed CCR and the role of CcpA in it have not yet been elucidated.

In the present study, we focused on the carbohydrate catabolism and its transcriptional regulation in *L. oligofermentans* type strain LMG 22743^T^ [1] as an example of an obligate heterofermentative *Lactobacillus*. For this, the complete genomic sequence of *L. oligofermentans* LMG 22743^T^ was provided [ENA: LN898144-LN898145] and manually annotated to study RNA-seq based time course transcriptomes during growth on glucose, ribose and xylose. In addition, phenotypical tests related to carbohydrate utilization, electron acceptors usage and respiration were performed. To gain insights into the transcriptional regulation of the catabolism of different carbohydrates, differential expression analysis was done between three different transcriptomes for three time points and the upstream regions of co-regulated genes were searched for the occurrence of common transcription factor binding sites (TFBS).

## Methods

### Genome sequencing and annotation

DNA was isolated from *L. oligofermentans* LMG 22743^T^(henceforth simply *L. oligofermentans*) grown in de Man-Rogosa-Sharpe (MRS) broth with 2 % xylose using the modified method [15] of Pitcher et al. [16]. The genomic DNA was mechanically sheared with a needle and a fosmid library was constructed using the CopyControl™ Fosmid Library Production Kit (Epicentre, Madison, WI, USA). Fosmids were grown in 2 ml deep-well plates and purified using the Montage BAC purification kit (Millipore, Billerica, Ma, USA) or the CosMCPrep (Agencourt, Beverly, Ma, USA). Fosmid end sequencing was done using BigDye Chemistry v.3.1 and analyzed on an ABI 3730 Sequencer (Applied Biosystems, Foster City, Ca, USA). Altogether 1,344 clones were end sequenced, generating 853,918 bp (ca. 0.5 × genome coverage).

A 454-shotgun sequencing library was generated from 10 μg of nebulized genomic DNA using a library preparation kit (454 Life Sciences/Roche, Branford, CT, USA). The library was amplified by emulsion PCR using an emulsion PCR kit (454 Life Sciences/Roche) and sequenced on a Genome Sequencer GS20 (Roche). A total of 225,045 sequences were generated corresponding to 24.5 Mb (13.3 × genome coverage). The sequences obtained were assembled using Newbler (454 Life Sciences/Roche). Contigs were co-assembled with the fosmid end sequences and edited using Gap4 from the Staden Package [17]. Gap closure was done by PCR and sequencing of the product or direct sequencing on selected fosmids.

Genome annotation was done analogously as in our previous study [18]. Briefly, coding DNA sequences (CDSs) were determined using EasyGene [19] and Prodigal [20], and gene functions were assigned using RAST [21] and PANNZER [22]. The CRISPRFinder [23], BAGEL2 [24], CW-PRED [25], and PHAST [26] programs were utilized to predict CRISPR/Cas systems, bacteriocins, LPxTG motif-containing proteins, and prophages, respectively. The automatic predictions were manually reviewed based on the presence of potential ribosomal binding sites, similarity searches, multiple-sequence alignments and bibliomic data. Finally, gene content was checked for the presence of all core functions [27] by classifying genes into functional COG categories using RPS-BLAST.

### Phenotypic analyses

Carbohydrate/carbon source utilization profiles of *L. oligofermentans* were tested by the API 50CH identification system (bioMeriéux, Marcy l'Etoile, France), and PM1 and PM2A Phenotype MicroArrays (BIOLOG, Inc., CA, USA) according to the manufacturer's instructions. In addition, *L. oligofermentans* was grown with selected carbon sources (glucose, N-acetylglucosamine, maltose, ribose, xylose, 2-deoxyribose, inosine, pyruvate, dihydroxyacetone [DHA] and glycerol) (75 mM) anaerobically (Anaerogen, Oxoid, UK) and aerobically (2 ml of culture in a 20 ml tube, 250 rpm) in three replicates. Growth without the carbon source was used as the control.

To test the utilization of carbon sources as electron acceptors, *L. oligofermentans* was grown with xylose (28 mM) as a primary carbon source, and pyruvate, DHA, glycerol or 2-deoxyribose (75 mM) as prospective electron acceptors or inosine (75 mM) as a secondary carbon source aerobically and anaerobically in three replicates. Similar concentrations of primary and secondary carbon sources (fructose and malate, respectively) were used by Cavin et al. [28] to test for malate utilization. The low concentration of a primary carbon source and significantly higher concentration of a secondary carbon source can be important to detect the effect of a secondary carbon source on growth. The anaerobic and aerobic growths with xylose alone (28 mM) were used as the controls. Acetoin and/or diacetyl (henceforth acetoin/diacetyl) production was detected using colorimetric Voges-Proskauer assay [29].

To test for respiration, *L. oligofermentans* was grown with glucose, ribose or xylose (50 mM) only, and with the addition of heme (2.5 μg/ml, in ethanol, Sigma-Aldrich, USA) or menaquinone (1.0 μg/ml, in 0.05 M NaOH, Sigma-Aldrich, USA) or both, heme and menaquinone, aerobically in six replicates. The growth without the carbon source was used as the control.

All the growth experiments were done in an MRS liquid medium without citrate, where glucose was substituted with the carbon sources of interest referred to above. Cultures were grown for 48 h at 25 °C with catalase (1000U/ml, Sigma-Aldrich, USA) to exclude the inhibitory effect of hydrogen peroxide on the growth. Hydrogen peroxide is produced by *L. oligofermentans* and its level is especially elevated during aerobic growth (data are not shown). The growth was measured as sample optical density at 600 nm [OD_600_]. The average values were calculated across the replicates. Two-tailed t-test’s *p-values* were calculated to assess the statistical significance of the observed differences.

For all the growth experiments *L. oligofermentans* was precultured for 24 h under the same conditions (medium, carbon source, atmosphere, temperature, etc.) used for the above experiments, followed by re-inoculation (initial OD_600_ ~ 0.01) into the fresh medium.

### RNA extraction, sequencing and differential expression analysis

*L. oligofermentans* was inoculated (OD_600_ of inoculated culture ~ 0.001 or initial cell density ~ 1 × 10^5^ CFU/ml) into a modified malo-lactic differential (MLD) medium [28] without malic acid, cellulose and bromocresol green, and with the concentration of a sole carbon source (either glucose, ribose or xylose) being 50 mM and pH adjusted to 5.2. The cells were adapted to growth on a certain carbon source by passing them through a medium with the same carbon source for at least 5 generations. The cultures were grown in three replicates per carbon source at 25 °C under microaerobic conditions (250 ml bottles with 200 ml of liquid medium, no shaking). The OD_600_ values were measured once an hour/two hours between 17 and 31 h of growth. In addition, viable cell counts were determined (using plate cultivation for 5 days on MRS medium with xylose instead of glucose, 25 °C, anaerobic atmosphere) to ensure that they correlated with the OD_600_ values. Samples were taken from each replicate at three time points 20, 24 and 30 h. RNA extraction and RNA sequencing libraries construction were done as in our previous study [18]. Briefly, cell metabolism and RNAase activity were inhibited by treatment with a cold 10:1 mixture of ethanol-phenol, samples were centrifuged and immediately frozen. The cells were disrupted by a mixer mill and RNA was extracted by the Rneasy Plant Mini Kit (Qiagen) with DNAase treatment. Ribosomal RNAs were omitted from the total RNA and RNA-seq libraries were produced using the Ovation RNA-seq System V2 (NuGEN). The dsDNA obtained was sheared using sonication, followed by polishing the fragments obtained with T4 DNA polymerase and ligating the adapters for SOLiD sequencing. Libraries (overall 27: three carbohydrates × three time points × three replicates) were size selected and thereafter sequenced in two runs with six lanes overall using SOLiD 5500XL (Life technologies, Foster City, Ca, USA) to produce 75 bp single-end reads.

Lifescope software (Life Technologies) was used for mapping and counting the RNA-seq reads, as well as for generation of the normalized read counts RPKM (Reads Per Kilobase of gene per Million mapped reads). The percentages of reads that were mapped to the *L. oligofermentans* genome varied from 23 to 87 % for different samples, and were identical between two sequencing runs. Approximately, half (44–52 %) of the mapped reads in each sample belonged to the protein-coding genes, which were used for further analysis. Four samples (libraries) that had a low number of CDS-mapped reads (<200,000) were discarded to avoid introduction of higher variability to the data especially for low expressed genes, leaving only two replicates per some conditions (combination of carbon source and time point). The number of CDS-mapped reads in samples used for the further analysis ranged from 0.76 to 13.84 million per sample (Additional file 1: Table S1). Hierarchical clustering of samples based on Euclidean distances and principle component analysis (PCA) was done using R functions as described in the manual for DESeq2 package on rlog-transformed data [30]. As the result, replicates belonging to the same carbon source clustered together at all time points on both heatmaps and PCA plots. This suggests that biological replicates for the same carbohydrate show higher similarity than samples obtained during growth on different carbohydrates, which indicates good quality of the RNA-seq data that were obtained and mapped.

Differential expression analysis for three pairs of transcriptomes, obtained during growth on different carbon sources (glucose-ribose, glucose-xylose and ribose-xylose), was performed independently for each time point (20, 24 and 30 h) using R package DESeq2 [30]. Default parameters were used. The genes were considered to be differentially expressed (DE) if their adjusted *p-value* was less than or equal to 0.05 and absolute log_2_ fold changes (FCs), obtained from DESeq2 output, were higher than or equal to 1 (commonly used parameters for differential expression analysis, e.g. [31]). The information on processed RNA-seq data (raw read counts, RPKMs) and differential expression analysis (adjusted *p-values* and log_2_FC) can be found in Additional file 2.

### Motif discovery and search

Upstream regions (in relation to the translational start site) of *L. oligofermentans* genes were extracted using a modified script, obtained from this study [32]. Upstream regions were extracted up to 300 nucleotides, should have had minimal length of 50 nucleotides and should not have overlapped with other CDSs. For each time point, co-regulated groups of genes were formed from genes, upregulated or downregulated in each pairwise comparison between three growth conditions (therefore six groups), and also genes, upregulated or downregulated in one growth condition in comparison with two others (also six groups). The next steps, including motif discovery search, enrichment analysis and querying the databases of TFBS, were done using the tools from MEME suite [33]. The upstream regions of each group of co-regulated genes were searched for commonly occurring motifs with *meme* tool using the following settings: minimum and maximum motif lengths were 6 and 50 nucleotides, respectively; the maximum number of different motifs obtained during the search was set to three; any number of repetitions of each motif per upstream region was allowed. The motifs discovered were tested for enrichment within the group of co-regulated genes in comparison with all the upstream regions extracted from the genome. This was done using the *ame* tool (method - one-tailed Fisher’s exact test). For this, the profiles of the motifs discovered were obtained from the *meme* output. During enrichment analysis, the genome nucleotide frequencies were taken into account in the form of a background hidden Markov model (HMM), built using *fasta-get-markov* script from the MEME suite. Selected enriched motifs were used to query the TFBS database using the *tomtom* tool (genome background HMM was taken into account). The database comprised the combination of the PRODORIC [34] and RegTransBase [35] databases that contain literature-based and manually-curated prokaryotic TFBS. For verification, *Lactobacillaceae*-specific TFBS profiles of CcpA, XylR, RbsR and Rex, as well as the *Bacillales*-specific Rex TFBS profile, were added to the database. These profiles were built using *sites2meme* script based on motif sequences obtained from the RegPrecise database [36] of manually-curated inferences of regulons in prokaryotes. The database itself did not contain built TFBS profiles, which made it impossible to directly search against it. In turn, *Lactobacillaceae*-specific TFBS profiles of CcpA, XylR, RbsR, as well as the Rex-like binding site profile, obtained from *L. oligoferementans* co-expressed genes (see Results), were used to search all the extracted gene’s upstream regions for the occurrence of CcpA, XylR, RbsR and Rex-like TFBS using the *fimo* tool with the *q-value* (adjusted *p-value*) threshold of 0.05 and with the account of genome background HMM.

## Results

### General genome characteristics

The complete genome of *L. oligofermentans* LMG 22743^T^ [ENA: LN898144-LN898145] included one 1,801,673-bp circular chromosome (Additional file 1: Figure S1) and one 43,810-bp circular plasmid. The overall GC content of the chromosome was low and equals 35.6 %. The numbers of the predicted protein-coding genes were 1,722 (including four putative pseudogenes) on the chromosome and 63 on the plasmid. In addition, the chromosome encoded 5 rRNA operons, 55 tRNA genes and 1 tmRNA gene. The putative functions, assigned to ~ 85 % of CDSs, were manually reviewed. The chromosome possessed only one DNA restriction-modification system of type II (LACOL_1094-1095) and one complete prophage region containing 55 genes (LACOL_0790-0844). One CRISPR/Cas system of type IIA was identified in the genome. It consisted of Cas-genes *cas9*, *cas1*, *cas2*, *csn2* (LACOL_0188-0191) and a repeat-spacer array, located downstream (start position: 207463; end position: 209479). Based on the predicted metabolic pathways, *L. oligofermentans* had a complete gene set for the phosphoketolase pathway, while it is missing the key genes for glycolysis - 6-phosphofructokinase *pfkA* and fructose-bisphosphate aldolase *fba*. This prediction confirmed that *L. oligofermentans* is an obligate heterofermentative LAB. Pyruvate could subsequently be fermented through pyruvate dehydrogenase, L- and D-lactate dehydrogenases and acetoin/diacetyl production pathways that were present in the genome, while acetyl-CoA could be fermented to either acetate or ethanol. The genome also possessed the components of an electron-transport chain, namely, *cydABCD*-encoded cytochrome *bd* quinol oxidase (LACOL_0071-0074), putative NAD(P)H dehydrogenase *ndh* (LACOL_0077) and putative NAD(P)H:quinone oxidoreductases *qor1* (LACOL_0099) and *qor2* (LACOL_0125). Protein products of *ndh*, *qor1* and *qor2* did not contain transmembrane regions. However, *ndh* and *qor1* were located in the vicinity of respiration-related genes. The latter might indicate their relation to the electron-transport chain. Typical to LAB, *L. oligofermentans* was missing a heme biosynthesis pathway and contains only a partial menaquinone biosynthesis pathway [37, 38]. The genome lacked biosynthesis pathways for a half of proteinogenic amino acids. Instead, it encoded approximately 11 amino acid transporters. All the genes required for the *de novo* purine and pyrimidine nucleotide biosynthesis were present in the genome. *L. oligofermentans* was particularly enriched in putative adhesins, having eight LPxTG-like motif-containing proteins and 14 anchorless candidates for adhesins, ten of which were serine-rich proteins (Additional file 1: Table S2). Based on the presence of the meso-diaminopimelate recognition motif (^412^DNPR^415^) in *murE* gene (LACOL_1711) [39], the peptidoglycan peptides should contain meso-diaminopimelatic acid in the third position, which was shown to be present in other members of the *L. vaccinostercus* group [7, 40, 41], but otherwise is not common for lactobacilli. The presence of Phe^260^ in the active site of D-Ala--D-Ala ligase *ddl* (LACOL_1290) indicates the resistance to vancomycin [42], which was confirmed by us (data not shown). Bacteriocin genes were not identified.

### Utilization of different carbon sources and their catabolic pathways

**Fig. 1:**
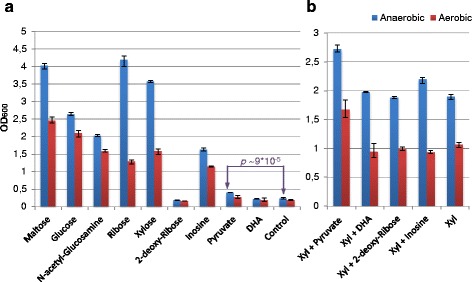
*L. oligofermentans* LMG 22743^T^ growth on different carbon sources (**a**) and xylose supplemented with secondary carbon sources (**b**). The experiments were done anaerobically (*blue bars*) and aerobically (*red bars*). The height of the bars corresponds to the average value across three replicates, and error bars show range of the values. Notation ‘Xyl’ means xylose; ‘DHA’ means dihydroxyacetone; ‘*p*’ is a two-tailed t-test’s *p-value*

To identify and verify carbohydrates/carbon sources that can support the growth of *L. oligofermentans* LMG 22743^T^, several carbohydrate utilization experiments were performed (Table 1). All the tests were positive for pentose utilization (ribose, xylose and arabinose), while for hexoses and other carbon sources, the results differed. Whereas in the API 50CH test, this strain produced acid from glucose, maltose and N-acetylglucosamine, BIOLOG phenotype microarrays showed a negative result for these substances. On the other hand, phenotype microarrays were positive for pyruvate, DHA, 2-deoxyribose and nucleosides. Finally, the actual growth experiments with the selected carbon sources confirmed the ability of this strain to grow with glucose, maltose, N-acetylglucosamine and inosine, while 2-deoxyribose and DHA did not support growth (Fig. 1a). For pyruvate minor (FC ~ 1.7) but statistically significant (*p-value* 9 × 10^−5^) anaerobic growth promotion was observed in comparison with the control. The aerobic atmosphere caused a considerable decrease in biomass on all the growth-supporting carbon sources (even in the presence of catalase), changing the relative growth efficiencies on different carbon sources so that anaerobically the highest biomass was observed on maltose, ribose and xylose, while aerobically the most efficient growth occurred on maltose and glucose. As can be seen, the biomass on maltose was among the highest biomass values under both anaerobic and aerobic conditions. Peculiarly, the presence of oxygen impaired the growth on pentoses (xylose and ribose) most of all. Phenotype microarrays measure only the level of cell redox activity and not the growth or fermentation directly [43]. Therefore the positive result might also indicate the use of the carbon source as an electron acceptor. Only the addition of pyruvate to the medium, containing xylose as a carbon source, increased biomass by ~ 50 % in comparison with the control both aerobically and anaerobically (*p-values* 5 × 10^−5^ and 0.003, respectively), while DHA and 2-deoxyribose did not have any effect (Fig. 1b). At the same time, the co-fermentation of two growth-supporting carbon sources (xylose and inosine) did not have a significant effect on growth, indicating that pyruvate-induced growth promotion was most probably attributed to its electron acceptor properties. The production of acetoin/diacetyl as an indirect indication of the use of external electron acceptors [44] occurred also only in the presence of pyruvate (in addition to xylose) under anaerobic conditions.

**Table 1:** The results of the carbohydrate/carbon source utilization tests for *L. oligofermentans* LMG 22743^T^

Carbon source	API 50CH^a^			BIOLOG phenotype microarrays^b^	Growth experiments^c^
	Koort et al. [1]	Tohno et al. [7]	Present study		
D-Glucose	(+)		+	−	+
D-Fructose	−	(+)	−	−	
D-Mannose	−	+	−	−	
D-Galactose	−	(+)	−	−	
D-Gluconate	(+)	+	(+)	−	
N-Acetyl-D-Glucosamin	(+)		(+)	−	+
D-Xylose	+		+	+	+
D-Ribose	+		+	+	+
L-Arabinose	+		+	+	
2-Deoxy-D-Ribose				+	−
Maltose	(+)		+	−	+
Pyruvate				+	(+) ^d^
Glycerol	−			−	−
Dihydroxy-acetone				+	−
Inosine, Adenosine, Uridine				+	+^e^

^a^API 50CH identification system measures acid production from carbon sources: +, positive reaction, −, negative reaction; (+), week or delayed reaction; empty field if not tested

^b^BIOLOG phenotype microarrays measure the level of cell redox activity: +, the score > 50; −, the score < 18 (negative control = 18)

^c^Growth experiments were done in an MRS medium without citrate anaerobically and aerobically: +, the OD_600_ FC > 5 in comparison with the control; −, no growth observed

^d^For pyruvate very weak (FC ~ 1.7), but statistically significant anaerobic growth promotion was observed in comparison with the control

^e^Tested only for inosine

Catabolic pathways and transport systems were predicted to be present in the genome for all the above-mentioned carbohydrates/carbon sources. In addition, *L. oligofermentans* possessed genes for the transport/catabolism of fructose, mannose, galactose, gluconate, L-ascorbate, N-acetylneuraminate, lactose, glycerol, 1,4-beta xylan- and L-arabinan-derived oligosaccharides (Additional file 1: Table S3). Only four phosphotransferase systems, transporting mannose *manXYZ* LACOL_0353-0355 (and possibly glucose, fructose and other hexoses [45]), N-acetylglucosamine *nagE* (LACOL_0524), ascorbate *ulaABC* (LACOL_0118-0120) and unidentified sugar (LACOL_0103-0106), were present in the genome.

### Comparison of transcriptome responses during exponential growth on glucose, ribose and xylose

**Fig. 2:**
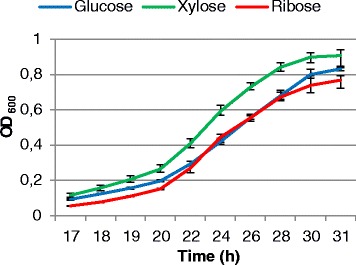
*L. oligofermentans* LMG 22743^T^ growth on glucose, ribose and xylose. The values are averages of three biological replicates and error bars show range of the values

**Fig. 3:**
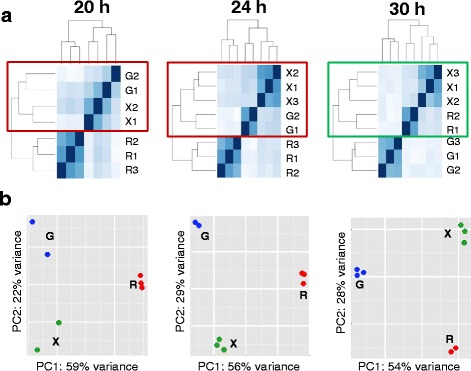
Similarity-based clustering (**a**) and PCA plots (**b**) for RNA-seq samples from three time points. Hierarchical clustering is based on Euclidean distance. Time points are taken at the beginning (20 h), middle (24 h) and end (30 h) of the exponential growth phase. Designations used: G – glucose, X – xylose, R- ribose. Different replicates for each carbon source are numbered (e.g. G1, G2, G3 etc.)

According to the growth curves of *L. oligofermentans* on three different carbohydrates (Fig. 2) under microaerobic conditions, growth was slightly faster on xylose than on glucose and ribose. Samples were taken at three time points (20, 24 and 30 h), which corresponded to the beginning, middle and end of the exponential growth phase, respectively. No plasmid-encoded genes were expressed, suggesting that the plasmid might have been lost during cultivation. To assess the level of differences/similarities between samples at each time point, we performed hierarchical clustering of samples based on the Euclidean distances between them. As Fig. 3a shows, the relative clustering of transcriptomes related to growth with the different carbohydrates changed during the course of time. Unexpectedly, in the beginning and the middle of the exponential growth phase (20 and 24 h), xylose transcriptome clustered together with glucose transcriptome, but at the end of the phase (30 h) it became more similar to ribose transcriptome. The same trend was seen on the PCA plots (Fig. 3b). For the 20 and 24 h samples, the first principal component (PC1), which contained 59 and 56 % of variance, respectively, clearly separated ribose samples from other samples. The second principal component (PC2), contributing to 22 and 29 % of variance, respectively, discriminated between all three transcriptomes with the larger separation between glucose and xylose samples. Nevertheless, at 30 h, the glucose samples became clearly separated from other transcriptomes by PC1 (54 % variance), while the PC2 (28 % variance) discriminated between all the transcriptomes with the largest separation between xylose and ribose samples.

Differential expression analysis was performed pairwise between three different transcriptomes (hereafter referred as pairwise comparisons) at each time point independently. Furthermore, based on the pairwise comparisons, new groups of genes with different expression patterns were identified (hereafter referred as triplewise comparisons). These groups included genes that showed significantly different expression during growth with one carbon source in comparison with two other carbon sources (three groups) or genes that had significantly different expression between all transcriptomes (one group) (Additional file 1: Table S4). Overall, the total numbers of DE genes (Table 2 and Additional file 1: Figure S2, containing Venn diagrams) reflected the detected relationships between the different transcriptomes. At 20 h, the number of DE genes between glucose and xylose transcriptomes was the lowest out of three pairwise comparisons, while the number of genes that showed significantly different expression during growth on ribose in comparison with glucose and xylose was the highest. Consequently, the number of genes having distinct expression in ribose transcriptome in comparison with glucose and xylose transcriptomes was much higher than for other groups of DE genes in the triplewise comparison. Later on, at 30 h, the number of DE genes between xylose and ribose was the lowest and the number of genes that had different expression level in glucose in comparison with two other transcriptomes was the highest. This was also reflected in the number of genes that had different expression when growing on glucose in comparison with pentoses, with the number being almost twice as high as for other groups in triplewise comparisons. Differentially expressed genes were subdivided more or less equally between upregulation and downregulation in almost all the comparisons and groups of DE genes.

**Table 2:** The number of differentially expressed genes for pairwise and triplewise comparisons between growth conditions

	20 h			24 h			30 h		
	No. of genes	Up, %	Down, %	No. of genes	Up, %	Down, %	No. of genes	Up, %	Down, %
Pairwise comparisons^a^									
G/R	268	53	47	374	46	54	292	47	53
G/X	61	57	43	145	34	66	279	45	55
X/R	289	52	48	260	56	44	197	58	42
Triplewise comparisons^b^									
X ≠ G ≈ R	13	46	54	36	92	8	68	66	34
R ≠ G ≈ X	140	48	52	121	62	38	65	49	51
G ≠ R ≈ X	29	66	34	52	50	50	114	45	55
G ≠ R ≠ X	12			17			7		

Other designations used: *Up* upregulation, *Down* downregulation

^a^ *G* indicates glucose, *R* ribose, *X* xylose. Notation G/R means the comparison between glucose and ribose transcriptomes, where up- and downregulation is given for glucose transcriptome relative to ribose transcriptome

^b^X ≠ G ≈ R designates the group of genes that have distinct expression in xylose transcriptome in comparison with glucose and ribose transcriptomes, where the last two have relatively similar expression; up- and downregulation is given for xylose transcriptome relative to glucose and ribose transcriptome; G ≠ R ≠ X – the group of genes that have distinct expression levels in all three transcriptomes

### Differential expression of carbohydrate/carbon source catabolic pathways and transporters

**Fig. 4:**
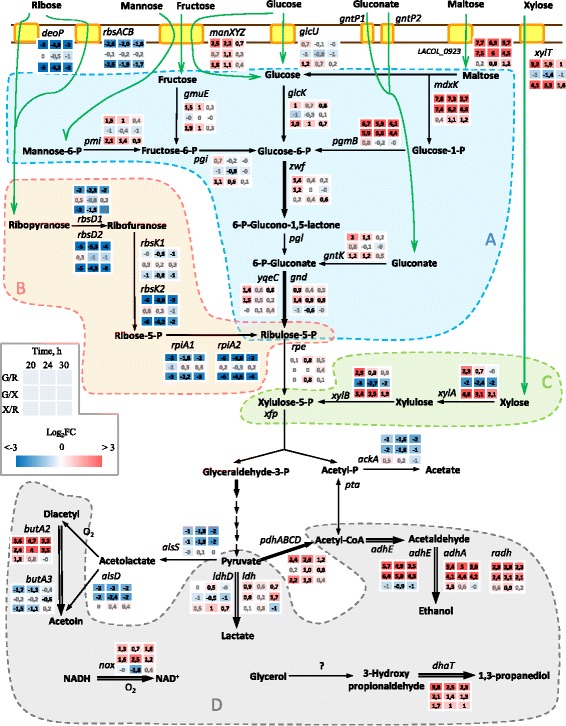
Differential expression of the genes involved in carbohydrate transport/catabolism, fermentation and NAD(P)H re-oxidation. Colored areas: blue (**a**) catabolism of hexoses and maltose, pink (**b**) ribose catabolism, green (**c**) xylose catabolism, grey (**d**) NAD(P)H re-oxidation. Bold arrows indicate NAD(P)H-producing reactions, white arrows – NAD(P)H-re-oxidizing reactions, yellow boxes represent transporters and green arrows – transport across the cell membrane. Notation ‘G/R’ means the ratio of normalized read counts between glucose and ribose samples. The 3 × 3 matrices for each enzyme/transporter gene contain values log_2_FC for pairwise comparisons between three transcriptomes for three time points, as indicated in the legend. The log_2_FC values are shown in black if the change was statistically significant (adjusted *p-value* < = 0.05) and in gray if it was not statistically significant

The growth on different carbohydrates (glucose, ribose and xylose) induced the expression of the corresponding catabolic pathways and transporters (Fig. 4). Two putative transporters were predicted to transport glucose: putative glucose uptake permease (LACOL_1336) and PTS mannose-specific transporter *manXYZ* (LACOL_0353-0355). Of these, only the PTS transporter was clearly upregulated on glucose in comparison with xylose and ribose at 20–24 h, while the permease seemed to be slightly upregulated in xylose samples. This suggests that *manXYZ,* and not the permease, is probably the main glucose transporter. It is notable that there was also significant overexpression of *manXYZ* in xylose cultures in comparison with ribose, and the expression levels of *manXYZ* decreased with time on both glucose and xylose (Additional file 1: Figure S3). Expectedly, the genes involved in glucose oxidation as part of the phosphoketolase pathway were overexpressed during growth on glucose at 20–24 h. These genes included glucose-6-phosphate dehydrogenase *zwf* (LACOL_0233) and two phosphogluconate dehydrogenases, *gnd* and *yqeC* (LACOL_0230 and LACOL_0240) (Fig. 4a). Unexpectedly, glucokinase *glcK* (LACOL_0779) was upregulated on both glucose and xylose at 20–24 h. Noteworthy, a maltose catabolic operon (LACOL_0921-0923) was highly induced by glucose with log_2_FC ~ 5.9–7.8 at 20 h and ~ 4.1–5.7 at 30 h (Fig. 4a). According to the RPKMs values (Additional file 2), this operon was almost not expressed in ribose and xylose samples, while the expression on glucose was high, but decreased significantly with time. The extreme induction of the maltose transporter by glucose raises the possibility that this transporter could be involved in glucose transport. However, this has not been shown so far for its homologs in other bacteria [46].

Out of three predicted xylose-proton symporters *xylP1*, *xylP2* and *xylT* (LACOL_0262, LACOL_0266, LACOL_1587), *xylT* had the highest expression level on xylose (~20–26 times higher than the other two transporters at 20 h, [Additional file 2]), suggesting the major role of this transporter in xylose transport. The expression of *xylT* was the highest during the growth on xylose at 20–24 h, although it was also upregulated on glucose in comparison with ribose (Additional file 1: Figure S3). During the time course, the expression of *xylT* decreased both on xylose and glucose. Xylose catabolic genes *xylAB* (LACOL_0401-0402) showed a similar differential expression pattern to *xylT*, but its upregulation in xylose cultures was more pronounced (Fig. 4c).

Ribose catabolism/transport genes were arranged in two gene clusters (LACOL_0441-0447 and LACOL_0616-0619). Both clusters contained genes for ribokinases *rbsK1* and *rbsK2*, ribose-5-phosphate isomerases *rpiA1* and *rpiA2*, D-ribose pyranases *rbsD1* and *rbsD2*. In addition, the first cluster included ribose operon repressor *rbsR* and ribose ABC transporter *rbsACB*, while the second cluster harbored an additional secondary transporter, which showed 52 % identity with the permease *deoP* from *E.coli*, where it is a part of the deoxyribose catabolic cluster [47]. This suggests that this transporter might be involved in (deoxy)ribose transport. Except for *rbsR* and *rbsK1*, genes in both clusters were clearly overexpressed during growth on ribose in comparison with glucose and xylose at all time points, although the ribose-induced upregulation was stronger for the second cluster (Fig. 4b). Interestingly, the time course dynamics of gene expression on ribose was different for these two clusters. While the first cluster was expressed at a comparatively constant level, the second cluster was strongly downregulated with time (Additional file 2, and for the ribose transporters Additional file 1: Figure S3). Comparing the expression levels of the isoforms from ribose catabolic clusters, it can be seen that *rbsD2* was much higher expressed than *rbsD1* (in the media containing ribose) at all times, whereas *rbsK*(s) and *rpiA*(s) had comparable expression levels in both clusters in the beginning (20 h) and much higher levels in the first cluster later on (24–30 h) (Additional file 2).

Nucleosides provide a source of carbon in the form of ribose in some foods such as meat. Peculiarly, genes involved in nucleoside degradation, were either evenly expressed on different carbon sources or even upregulated on glucose and xylose in comparison with ribose at the beginning of the exponential growth phase. The second group included an operon *deoDBC* (LACOL_1574-1576), consisting of purine nucleoside phosphorylase, phosphopentomutase and deoxyribose-phosphate aldolase, respectively, and inosine-uridine preferring nucleoside hydrolase (LACOL_0326).

### Differential expression of fermentation, NAD(P)H re-oxidation and respiration related genes

The expression of the genes involved in the fermentation of pyruvate and acetyl phosphate, produced as a result of the carbohydrate catabolism through the phosphoketolase pathway, was largely affected by the carbohydrate present in the growth media (Fig. 4). Catabolism of pentoses (ribose or xylose) led to the continuous (20–30 h) and significant upregulation of acetoin/diacetyl formation from pyruvate (acetolactate synthase *alsS* [LACOL_0671] and acetolactate-decarboxylase *alsD* [LACOL_0672]) and acetyl phosphate fermentation to acetate (acetate kinase *ackA* [LACOL_1217]). The expression of two lactate dehydrogenases was carbohydrate-dependent. L-lactate dehydrogenase *ldh* (LACOL_1517) had mildly higher expression in glucose samples, while D-lactate dehydrogenase *ldhD* (LACOL_1076) in xylose samples. Finally, the pyruvate dehydrogenase pathway, encoded by *pdhDCBA* (LACOL_1150-1153), exhibited a differential expression pattern that reflects the above-mentioned transcriptome clustering. At 20 h, the expression levels were similar on glucose and xylose, and higher than on ribose, although over the time course they became similar for ribose and xylose, and lower than for glucose.

The ethanol branch, involved in NAD(P)H re-oxidation and catalyzed by aldehyde-alcohol dehydrogenase *adhE* (LACOL_0255), was highly upregulated (log_2_FC ~ 5.7–6.4 at 20 h and ~ 3.5–4.3 at 30 h) during the catabolism of glucose. Furthermore, the genome possessed two additional alcohol dehydrogenases that might be involved in the production of ethanol and which were also highly induced by glucose. These included *adhA* (LACOL_0760) [48] and the short-chain dehydrogenase (LACOL_0253), which showed 82 % identity to R-specific alcohol dehydrogenase *radh* from *Lactobacillus brevis*. While *adhE* and *adhA* were downregulated in time during glucose catabolism, *radh* was upregulated instead. Besides alcohol dehydrogenases, the growth on glucose led to elevated levels of other NAD(P)H re-oxidizing enzymes (Fig. 4d). These included NAD(P)H oxidase *nox* (LACOL_1537), one of the three diacetyl reductases *ButA2* (LACOL_1011) and 1,3-propanediol dehydrogenase *dhaT* (LACOL_0222). The last gene encodes the second enzyme of the 1,3-propanediol biosynthesis from glycerol, for which the first enzyme, glycerol dehydratase, was not identified in the genome.

Respiration-related genes were also found to be DE depending on carbon-source: cytochrome biosynthesis operon *cydABDC* showed higher transcript levels on xylose at 30 h, while *ndh*, *gor1* and *qor2* were upregulated on both ribose and xylose at 24–30 h. While testing for respiration (Additional file 1: Figure S4), the addition of menaquinone alone caused a slight increase in biomass during aerobic growth on glucose and xylose. However, supplementation with heme in addition to menaquinone did not lead to further growth promotion, suggesting that *L. oligofermentans* did not respire under these conditions.

### Transcriptional regulation of genes with similar differential expression profiles

To shed light on the regulatory mechanisms underlying the observed transcriptome changes depending on the carbon source, we searched *de novo* the upstream regions of co-regulated genes to discover commonly occurring motifs, tested for their enrichment and queried the databases of TFBS with the identified motifs (Table 3).

**Table 3:** Motifs discovered *de novo* ^a^ and enriched in the upstream regions of the co-regulated genes

Groups of co-regulated genes^b^	Enrichment *p-value*	No. of upstream regions, total	No. of upstream regions with the motif(s)	TFBS database match [database] (*q-value* ^c^)
G > R, 20 h	6.58e-6	86	31	*Lactobacillaceae* CcpA [RegPrecise] (1e-07)
				*Bacilli* CcpA [RegTransBase] (1e-06)
X > R, 20 h	1.76e-10	90	38	*Lactobacillaceae* CcpA [RegPrecise] (2e-08)
				*Bacilli* CcpA [RegTransBase] (4e-07)
G ≈ X > R, 20 h	1.04e-5	42	21	*Lactobacillaceae* CcpA [RegPrecise] (1e-09)
				*Bacilli* CcpA [RegTransBase] (2e-07)
G > X, 20 h	1.32e-5	23	11	*Bacillales* Rex [RegPrecise] (9e-08)
				*Lactobacillaceae* Rex[RegPrecise] (1e-07)
				*Bacillus* Fnr [PRODORIC] (2e-03)
G > X, 24 h	1.92e-5	31	13	*Bacillales* Rex[RegPrecise] (2e-09)
				*Lactobacillaceae* Rex [RegPrecise] (7e-09)
				*Bacillus* Fnr [PRODORIC] (6e-05)

^a^ Sequence logos for the discovered motifs can be found in Additional file 1: Table S6

^b^ *G* indicates glucose, *R* ribose, *X* xylose. Notation G > R designates the group of genes that have higher expression during growth on glucose than on ribose; G ≈ X > R – the group of genes that has similar expression levels on glucose and xylose, which are higher than on ribose

^c^ *q-value* is an adjusted *p-value*, as given by *tomtom* tool from MEME suite [33]

The groups of genes overexpressed during growth on glucose and/or xylose in comparison with ribose in the beginning of the exponential growth phase (20 h) were significantly enriched in a motif that was similar to a CcpA- binding motif of *Lactobacillaceae* and *Bacilli*. Genes containing this motif in their upstream regions included, for example, genes involved in the catabolism and/or transport of fructose, mannose, glucose, gluconate, and xylose, as well as pyruvate dehydrogenase complex and central components of the PTS and the carbon catabolite control (CCC) system *ptsH* and *ptsI*. To discover more genes regulated putatively by CcpA, we scanned the upstream regions of all the genes in the genome for the *Lactobacillaceae*-specific CcpA- binding motif (obtained using RegPrecise database). As a result, 109 upstream regions containing this motif were found (Additional file 3). Interestingly, even at 20 h, when the concentrations of carbohydrate sources were at their highest in comparison with other time points (24 and 30 h), 54 (50 %) of the genes that had been predicted to have CcpA- binding motif in their upstream regions did not show differential expression in any pairwise comparisons. And yet, within the predicted CcpA- binding motif-containing genes that showed differential expression at 20 h (Additional file 1: Table S5), the prevailing groups of genes were those that had different expression levels on ribose in comparison with glucose (37) or xylose (45) or both glucose and xylose (25) samples. In these groups most of the genes were upregulated on glucose (81 %), xylose (75 %) or both (75 %), respectively. In addition to the genes upregulated on glucose and xylose in comparison with ribose described above, CcpA-binding motif adjacent genes included, among others, ribose catabolism/transport related genes, catabolism of N-acetylneuraminic acid, galactose/lactose, arabinose, aldehyde-alcohol dehydrogenase, glucose-6-phosphate dehydrogenase and CRISPR-associated protein *cas9* (Table 4)*.* CcpA (LACOL_1227) is the central component of CCC, which also includes phosphocarrier protein HPr (*ptsH,* LACOL_0614), phosphoenolpyruvate-protein phosphotransferase (*ptsI,* LACOL_0615) and HPr serine kinase/phosphorylase (*hprK,* LACOL_1394). All of which were expressed based on RNA-seq data. Genes *ccpA* and *hprK* did not show significant expression differences depending on carbon source at any time point, while *ptsH* and *ptsI*, as outlined above, were overexpressed on glucose and xylose at 20–24 h.

**Table 4:** Genes predicted to contain a CcpA- binding motif^a^ in the upstream regions

Gene locus_tag	Gene function/name	Log_2_FC, 20 h^b^			Motif start coordinate^c^	Motif sequence	Co-transcribed genes
		G/R	G/X	X/R			
Genes, upregulated on glucose and xylose							
LACOL_0264	Fructokinase *gmuE*	**1.46**	−0.4	**1.86**	−88	ATTAAAACGGTTACAA	
LACOL_0353	PTS system mannose-specific EIIAB component *manX*	**2.47**	0.49	**1.98**	−97	ATGAAAGCGTATTCAA	*manXYZ*
LACOL_0639	Mannose-6-phosphate isomerase *pmi*	**1.54**	−0.52	**2.06**	−58	TAGGAAGGGCTTACAT	
LACOL_0566	Gluconokinase *gntK*	**2.03**	0.84	**1.19**	−112	ATGGAATCGGTTGCTA	LACOL_0567 ^d^
					−95	TTGTAACCGATTTCCA	
LACOL_1326	Gluconate permease *gntP*2	**1.81**	−0.21	**2.02**	−155	TTGTTAACGGTTACAA	LACOL_1325 ^d^
LACOL_0266	Putative xylose-proton symporter *xylP2*	**1.6**	0.78	**0.82**	−130	TTGCAAGCGTTTACAA	LACOL_0265 ^d^
LACOL_0399	Aldose 1-epimerase *xylM*	**1.09**	−0.39	**1.47**	−144	ATACAAGCGCTTTCAT	*xylR* ^d^
					−46	ATTTAATCGCTTACAT	
LACOL_0400	Transcriptional xylose repressor *xylR*	1.19	−0.43	**1.62**	−239	ATGTAAGCGATTAAAT	*xylM* ^d^
					−141	ATGAAAGCGCTTGTAT	
LACOL_0401	Xylose isomerase *xylA*	**2.33**	**−2.3**	**4.63**	−90	GTGAAAGGGGTTGCAA	*xylA-xylB*
					−68	ATGTAAGCGTTATACT	
LACOL_1587	D-xylose-proton symporter *xylT*	**3.23**	−0.9	**4.13**	−139	TTGGCAGCGGTTTCAT	
					−72	CTGAAAGCGGTTACGC	
LACOL_0614	Phosphocarrier protein Hpr *ptsH*	**1.61**	0.26	**1.36**	−126	AATAAAACGTTTACAT	*ptsH-ptsI*
LACOL_1153	Pyruvate dehydrogenase E1 component alpha subunit *pdhA*	**2.54**	0.26	**2.28**	−60	AATAAAGCGCTTACAT	*pdhA-pdhB-pdhC-pdhD*
LACOL_0222	1,3-propanediol dehydrogenase *dhaT*	**3.81**	**2.11**	**1.7**	−41	TTGTAATCGCTTTAAT	LACOL_0223 ^d^
Genes, upregulated on ribose							
LACOL_0444	D-ribose pyranase *rbsD1*	**−2.32**	0.55	**−2.87**	−77	TTGAAAGCGGTTACTA	*rbsD1-rbsA-rbsC-rbsB*
LACOL_0616	Putative (deoxy)ribose permease *deoP*	**−4.59**	0.04	**−4.63**	−50	TTGTAAGCGGATTATT	
LACOL_0617	D-ribose pyranase *rbsD2*	**−4.57**	0.34	**−4.9**	−108	TTGCAATCGTTTCCAA	
LACOL_0618	Ribokinase *rbsK2*	**−5.55**	0.59	**−6.13**	−258	TTGGAAACGATTGCAA	*rbsK2-rpiA2*
Genes, upregulated on glucose							
LACOL_0233	Glucose-6-phosphate dehydrogenase *zwf*	**1.37**	**1.19**	0.18	−107	GTGTAACCGGTTTATT	
LACOL_0255	Aldehyde-alcohol dehydrogenase *adhE*	**5.69**	**6.37**	−0.68	−220	ATGTAAGCGATTACAA	
LACOL_0923	Sugar (maltose):cation symporter	**7.66**	**7.46**	0.2	−53	CTGTAATCGGTTACAT	LACOL_0923*-mdxK-pgmB*
Other genes (mostly equally expressed)							
LACOL_0188	CRISPR-associated protein *cas9*	0.56	−0.52	**1.09**	−67	ATGAAAGCGTTTAACC	
LACOL_0275	L-arabinose isomerase *araA*	0.81	−0.19	**1**	−79	TTGTAAGCGATTAACA	
LACOL_1004	N-acetylmannosamine-6-phosphate epimerase *nanE*	0.48	−0.13	0.61	−263	ATGTAAACGTTTTCTT	*nanE-nanA-nanR, nanK* ^d^
LACOL_1005	N-acetylmannosamine kinase *nanK*	0.6	−0.55	**1.14**	−36	AAGAAAACGTTTACAT	*nanE* ^d^
LACOL_0104	PTS system sugar-specific enzyme IIA component	0.4	0.3	0.1	−63	TTGTAAGCCTTTGCAA	LACOL_0104-LACOL_0106
LACOL_0218	Galactokinase *galK*	0.21	0.33	−0.12	−133	AATTAACCGTTTTCAT	*galK-galT-galR*
LACOL_0521	Beta-galactosidase large subunit *lacL*	0.39	−0.57	**0.97**	−63	TTGAAAGCGCTTTAAC	*lacL-lacM*
LACOL_1707	Beta-galactosidase *lacZ*	0.06	0.58	−0.52	−38	AAGAAAGCGCTTTCTA	*LacZ-*LACOL_1706-LACOL_1705

^a^ CcpA- binding motifs were found by scanning all upstream regions in the genome with the Lactobacillaceae-specific CcpA TFBS profile built using RegPrecise database

^b^ For the designations, such as G/R, see Table 2. Statistically significant changes are marked in bold

^c^ Motif start coordinate is given in relation to the predicted translational start site

^d^ The gene/operon upstream region is overlapped with the upstream region of the adjacent divergently oriented gene

The group of genes upregulated during catabolism of glucose in comparison with xylose at 20 h and 24 h was found to be enriched in a motif that had significant similarity (*q-values* ~ 2e-03 and 6e-05, respectively) to the *Bacillus subtilis* anaerobic regulator Fnr TFBS [PRODORIC:MX000005] [34] (Table 3) when the initial search against PRODORIC and RegTransBase databases was performed. Nevertheless, the literature search identified another potentially matching transcription factor, redox-sensing transcriptional repressor Rex [49], the binding site of which was not present in the databases outlined. When *Lactobacillaceae*- and *Bacillales*-specific Rex TFBS profiles, which were built from the corresponding TFBS sequences present in RegPrecise database, were included in the above mentioned databases, the enriched motif matched Rex TFBS with much higher similarity (*q-values* ~ 9e-08 and 2e-09 for the *Bacillales*-specific profile and 1e-07 and 7e-08 for the *Lactobacillaceae*-specific profile) than Fnr TFBS.

**Fig. 5:**
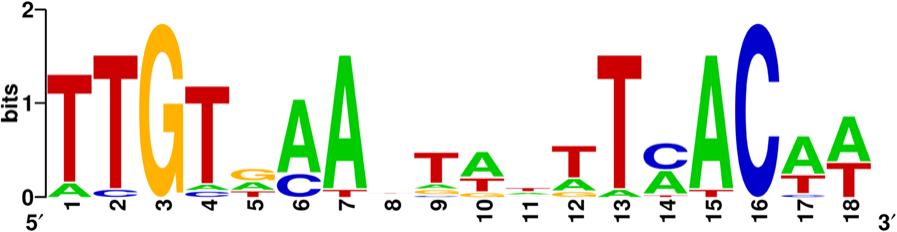
*L. oligofermentans* LMG 22743^T^ predicted Rex TFBS-like motif profile

To determine all the potential occurrences of the identified Rex TFBS-like motif in the genome, all the upstream regions were scanned with the motif profile discovered in the group of genes overexpressed on glucose in comparison with xylose at 20 h (Table 3). The motif was found in front of 20 genes. We noticed that many of the genes (12 out of 20) were upregulated on glucose in comparison with both xylose and ribose most of the time (Table 5). Curiously, all these genes seemed to be monocistronic. The motif profile (Fig. 5) derived from these genes only (Table 5) and, therefore, related to the upregulation on glucose in comparison with pentoses, was again much more similar to the binding site of the Rex regulator (*q-value* ~ 4e-09 for the *Lactobacillaceae*-specific profile and 6e-09 for the *Bacillales*-specific profile) than to the binding site of the Fnr regulator (*q-value* ~ 1e-04)*.* The motif represented the partial palindromic sequence of an approximate length of 18 nucleotides with conserved position at G3 and C16, probably required for pairing. The pairing also might be mediated by the AT pairs, formed by the A- and T- stretches, surrounding conserved G and C nucleotides. Six out of 12 genes (Table 5) were (putatively) involved in NAD(P)H re-oxidation (*adhE*, *adhA, radh*, *nox*, *butA2* and *dhaT*). All alcohol dehydrogenases and phosphogluconate dehydrogenase contained at least two such putative motifs in their upstream regions. The other six genes did not have a clear relation to redox balance maintenance, yet some of them could be involved in glucose catabolism: 6-phosphogluconate dehydrogenase (LACOL_0230) and RpiR family transcriptional regulator (LACOL_0239), which showed 27 % identity and 54 % similarity to HexR regulator involved in repression of glucose catabolism genes in *Pseudomonas putida* [50]. It is notable that none of the two lactate dehydrogenases that are also involved in NAD(P) + regeneration contained a Rex-like binding motif. The *L. oligofermentans* genome contained one gene for the redox-sensing transcriptional repressor Rex (LACOL_1407), which was not found to be differentially expressed in any pairwise comparisons at any time point.

**Table 5:** Genes containing Rex TFBS-like motif^a^ in the upstream regions and upregulated on glucose

Gene locus_tag	Gene function/name	Motif start coordinate^b^	Motif sequence	Log_2_FC^c^					
				20 h		24 h		30 h	
				G/R	G/X	G/R	G/X	G/R	G/X
LACOL_0123	Glycerol dehydrogenase *gldA*	−38	TCGTTAACTATTTCACAA	**1.5**	**2.7**	**1.4**	**2.0**	0.7	**2.1**
LACOL_0137	CoA-substrate-specific enzyme activase	−186	TTGTTCATTGAAACACAA	0.2	**0.9**	**0.7**	**1.7**	**0.7**	**1.3**
LACOL_0222^d^	1,3-propanediol dehydrogenase *dhaT*	−64	TTGTGAAATGCGTTACAA	**3.8**	**2.1**	**2.5**	**1.4**	**2.3**	**1.3**
LACOL_0230	Phosphogluconate dehydrogenase *gnd*	−103	TTGTGAATTTTTTAACTT	**0.9**	**1.4**	0.4	**0.9**	0.5	**0.9**
		−283	TTGAAAAACAATTCACTA						
LACOL_0239^e^	RpiR family transcriptional regulator	−56	ATGCAAATGTTTTCACAA	**2.6**	**2.2**	**1.0**	**1.2**	0.8	**1.4**
LACOL_0253^d^	R-specific alcohol dehydrogenase *radh*	−192	TTGTAAACTAGTTAACCT	**2.9**	**2.4**	**2.9**	**2.1**	**2.3**	**2.1**
		−67	TTGTGCTATAGTTCACAT						
LACOL_0255^d^	Aldehyde-alcohol dehydrogenase *adhE*	−48	TTGTGAATTAAGTAACAA	**5.7**	**6.4**	**4.9**	**5.8**	**3.5**	**4.3**
		−203	TTGTACACTAAATCACAA						
LACOL_0628	Peptidoglycan-binding protein	−171	ATGTGCACTTTTTAACAA	0.1	0.4	**1.6**	**1.1**	**2.2**	**3.1**
LACOL_0760^d^	Alcohol dehydrogenase *adhA*	−56	TTGTTAAGTATTTAACTT	**5.4**	**4.1**	**5.0**	**4.4**	**3.8**	**4.2**
		−105	TTGTTAATAATTTCACTT						
LACOL_1011^d^	Diacetyl reductase *butA2*	−34	TTGTGAATTAAATAACTT	**3.6**	**2.4**	**4.7**	**4.0**	**3.3**	**3.5**
LACOL_1481	Short-chain-enoyl-CoA hydratase	−65	TTGTGAAAATGATATCAT	**1.9**	**2.1**	**2.0**	**2.4**	**1.7**	**2.1**
LACOL_1537^d^	NADH oxidase *nox*	−44	TTGTAAAAGTTTTCACAA	**1.3**	**1.6**	**0.7**	**2.5**	**1.6**	**1.2**

^a^Rex TFBS-like motifs were found by scanning all upstream regions in the genome with the motif profile discovered in the group of genes overexpressed on glucose in comparison with xylose at 20 h (Table 3)

^b^Motif start coordinate is given in relation to the predicted translational start site

^c^For the designations, such as G/R, see Table 2. Statistically significant changes are marked in bold

^d^Genes, (putatively) involved in NAD(P)H re-oxidation

^e^LACOL_0239 shares its upstream region with LACOL_0240 (phosphogluconate dehydrogenase *yqeC*)

Apart from the motifs enriched in the groups of co-regulated genes, we also looked into the specific regulators of xylose (XylR [LACOL_0400]) and ribose catabolism (RbsR [LACOL_0441]), respectively. Overall, ten RbsR-like and two XylR-like binding sites were found in the genome (Additional file 3). Both ribose catabolic clusters were predicted to be regulated by RbsR and included the following transcriptional units: *rbsR*, *rbsK1*-*rpiA1, rbsD1ACB, rbsD2* and *rbsK2-rpiA2*. In addition, upstream regions of these operons, except *rbsR,* contained non-overlapping CcpA-binding sites (Additional file 3). Interestingly, the CcpA- binding motif (AATAAAACGTTTACAT), predicted in the promoter region of *ptsH-ptsI* operon (*q-value* 0.021), completely overlapped with the predicted RbsR- binding site (TAAATAAAACGTTTACATTA, *q-value* 0.048). As for two predicted XylR- binding sites, they were located in the promoter regions of xylose catabolism-related genes. One was between *xylM* and *xylR* and the other upstream *xylA-xylB* operon. In addition, these promoter regions (each) contained two non-overlapping CcpA- binding sites (Additional file 3). Gene *xylM* (LACOL_0399) was aldose 1-epimerase, which initially was annotated as maltose epimerase based on the similarity searches. But the presence of XylR- binding site in the upstream region of this gene and the vicinity of xylose catabolic genes suggest that this gene is probably involved in xylose catabolism. Consequently, it was also upregulated in response to xylose during 20–30 h.

## Discussion

The genetic background of *L. vaccinostercus* group has only recently started to be studied [4]. Even less is known about transcriptional regulation of the carbohydrate fermentation by this group of bacteria and obligate heterofermentative LAB in general since studies have focused mainly on homofermentative and facultative heterofermentative LAB. This paper presents the second complete and manually annotated genome (another is available for *L. hokkaidonensis* [4]) and the first study of carbohydrate catabolism-dependent transcriptome response for a member of *L. vaccinostercus* group. The number of coding genes (1,722) in *L. oligofermentans* genome is relatively small compared to other members of the *L. vaccinostercus* group (1,713–2,583 genes) [4, 5] [GenBank:AWTT00000000.1] and other lactobacilli (1,267–4,758 genes) [51]. Based on the ortholog prediction for the members of *L. vaccinostercus* group [4] (*L. wasatchensis* was not included), the genes, absent in *L. oligofermentans*, include, among others, those involved in amino acid biosynthesis. This could be explained by the *L. oligofermentans* association with a meat environment, which is rich in amino acids. Loss of amino acid biosynthesis genes is observed in other meat-borne lactobacilli, such as *L. sakei* and *L. algidus*, which were predicted to be auxotrophic for all amino acids [51].

Based on the predicted gene functions, this bacterium has the potential to utilize a multitude of different carbon sources. The previous study [7] and our results show that the real utilization capacity has been changing during the time from being limited to pentose utilization [1] toward an extended range of fermented carbon sources, which includes hexoses and maltose. We assume that the long-term cultivation of *L. oligofermentans* in the medium containing glucose could induce more efficient utilization of hexoses. Similar induction of hexose fermentation was observed for *L. vaccinostercus* [52]. These findings suggest that members of *L. vaccinostercus* show quite flexible carbohydrate catabolism, which can be adjusted, depending on the carbohydrate sources available in their habitats. This ability could be beneficial for *L. oligofermentans* in adaptation to grow in a meat environment, taking into account the higher concentration of free glucose than pentoses in meat. The considerably higher anaerobic growth efficiency on maltose in comparison with that on glucose is in agreement with the fact that obligate heterofermentative LAB prefer utilization of disaccharides to hexoses fermentation [53]. This is explained by the phosphorolytic cleavage of disaccharides, which generates glucose-6-phosphate without expense of ATP. The observed efficient catabolism of inosine, and probably other nucleosides, might offer a competitive advantage to *L. oligofermentans* in meat-derived products [10], where nucleosides are the main source of ribose moieties [54]. In facultative heterofermentative *Lactobacillus sakei,* nucleoside catabolic genes *deoDBC* were induced during the growth on ribose. However, unexpectedly, the opposite situation was observed in *L. oligofermentans,* the implication of which is not clear. Another carbon source, pyruvate, which is used for growth by some meat spoilage bacteria [55], supported the growth of *L. oligofermentans* very poorly, but the biomass increase and anaerobical production of acetoin/diacetyl in the presence of pyruvate, as well as the positive reaction for pyruvate in the BIOLOG phenotype microarrays, indicate its possible role as an electron acceptor. Generally, oxygen can also act as an electron acceptor through *nox*-mediated NAD(P)H oxidation and aerobic respiration. Still, the experiments demonstrated the inhibitory effect of oxygen on *L. oligofermentans* growth and did not reveal the evidence for the respiration ability in this bacterium. The menaquinone-associated biomass increase could be explained by its anti-oxidant properties. The glucose-induced overexpression of the aldehyde-alcohol dehydrogenase *adhE,* which was reported to possess an antioxidant activity in *E.coli* [56]*,* and possibly of the other alcohol dehydrogenases might explain the observed less pronounced inhibitory effect of oxygen on the biomass during growth on glucose in comparison with pentoses (ribose and xylose).

The higher similarity between glucose and xylose transcriptomes compared to the ribose transcriptome detected in the beginning of the exponential growth phase (20 h) is striking, since from both the chemical and metabolic points of view ribose and xylose are more alike. Both are pentoses and enter the phosphoketolase pathway nearly at the same level, only one reaction apart (Fig. 4). There seems to be a cross-induction both of glucose (putative hexose transporter *manXYZ*, glucokinase *glcK*) and xylose (xylose transporter *xylT* and xylose catabolic genes *xylAB*) transport/catabolic gene expression, by either glucose or xylose in comparison with ribose transcriptome (Fig. 4). Assuming that the original habitat for all the members of *L. vaccinostercus* group is phyllosphere, it can be speculated that similarities in transcriptome responses to growth with glucose and xylose could be caused by the ancient adaption of *L. oligofermentans* to utilize plant related polysaccharides, particularly hemicellulose. The latter consists mainly of glucose and xylose residues, and constitutes 30–55 % of plant cell walls [57]. The synergistic gene expression induction of the same pathways by glucose and xylose would probably result in more efficient fermentation of hemicellulose. The high induction of maltose catabolic operon expression only by glucose, and not pentoses, would presumably be beneficial during the fermentation of starch-rich plant material, which is eventually decomposed into glucose/maltose units. The later change (30 h) in similarity-based clustering of transcriptomes, when expression patterns during growth on xylose become more similar to those on ribose, might be caused by the decrease in carbohydrate concentrations in the media, as well as by differences in time course transcription regulation induced by the utilization of different carbohydrates. The changes in transcriptome similarities obtained during the course of time underline the importance of studying transcriptome at several time points to gain a more complete picture of transcriptome relationships.

As already noted, we did not detect glucose-induced repression of the catabolism of other carbohydrates, such as hexoses (mannose, fructose and gluconate) and xylose, as seen, for example, in *L. sakei* [58]. On the contrary, the stimulation effect was observed when comparing with ribose. A similar effect was noticed in *Leuconostoc gelidum* subsp. *gasicomitatum*, another obligate heterofermentative bacterium, where several disaccharide/hexose transporters were upregulated in glucose [59]. These findings suggest that transcriptional regulation of carbohydrate catabolism is different in obligate and facultative heterofermentative bacteria. The observed enrichment of CcpA-like binding sites in the upstream regions of genes, overexpressed on both glucose and xylose, indicates that CcpA could govern similar glucose and xylose transcriptome responses at the beginning of the exponential growth phase. Based on the predicted CcpA- binding motifs in the genome, CcpA is presumably involved in the global regulation of carbohydrate transport/catabolism and fermentation in *L. oligofermentans*. However, due to the fact that predicted CcpA-regulated genes exhibit different expression patterns (other than simultaneous upregulation on glucose and xylose), the exact mechanism of CcpA regulation, which might be a key to relaxed CCC observed in *L. brevis* [14] and *L. oligofermentans*, remains unclear and needs to be studied further, for example, by investigating the transcriptome profiles of knockout mutants of *ccpA* and, possibly, other CCR-related genes. The reverse genetics with this species has been proven to be very difficult. We have not been able to obtain knockouts by crossover recombination, even after excessive trials. Nevertheless, we speculate that the interplay with RbsR, XylR and other yet unidentified regulators, as well as the ambivalent repressive/activating nature of CcpA [60], could possibly explain the variety of observed expression patterns within CcpA-regulated genes.

As in other Firmicutes [61] (RegPrecise database), in *L. oligofermentans* XylR and RbsR repressors were predicted to be mainly involved in the regulation of xylose and ribose transport/catabolism genes, respectively, which is consistent with the strongest upregulation of these genes among other genes in the presence of xylose and ribose, respectively. In addition, these genes contain CcpA- binding sites in their upstream regions. Even though both ribose catabolic clusters are predicted to be regulated by both RbsR and CcpA, they have different time-dependent dynamics of expression changes, which in turn suggests that they could have different roles in ribose fermentation depending on the ribose concentration in the growth medium and/or growth phase. The complete overlap of RbsR- and CcpA- binding sites in the promoter region of the HPr-coding gene is peculiar, especially in the light of a study, which showed that serine phosphorylated HPr (HPr-Ser46-P) can interact not only with CcpA, but also with RbsR, and complex RbsR-HPr-Ser46-P is able to bind DNA, containing RbsR- binding site [62]. If this is the case, RbsR and CcpA would compete not only for HPr-Ser46-P, but also for the binding site in the promoter region of HPr. Similarly, this was suggested for the *rbs* operon in *Bacillus subtilis* [62]. This also implies that the ribose repressor RbsR might possibly be involved in CCC along with CcpA in *L. oligofermentans*. Interestingly, the growth with ribose induced opposite changes in HPr gene expression in *L. oligofermentans* (downregulation) and *L. sakei* (upregulation) [58]*.*

To enter the phosphoketolase pathway glucose, unlike pentoses, should be oxidized first. This is mediated by the oxidative branch of this pathway, which was, expectedly, upregulated during growth only with glucose. This creates a requirement for efficient re-oxidation of additional NAD(P)H, produced during glucose oxidation, to be able to grow on glucose [44]. It explains the extreme overexpression of genes involved in ethanol/alcohol production and other reactions coupled with NAD(P)H oxidation (Fig. 4d), when glucose instead of pentoses is present in the medium. On the contrary, in *L. sakei*, which ferments glucose through glycolysis, no changes in *adhE* expression were observed between growth on glucose and ribose [58]. Only one lactate dehydrogenase was slightly upregulated by glucose, which suggests that the adaptation to growth on hexoses in *L. oligofermentans* is mainly achieved through the activation of the ethanol branch and other NAD(P)H re-oxidizing systems, but not through pyruvate fermentation to lactate. Furthermore, the upregulation of the pyruvate dehydrogenase complex on glucose in comparison with pentoses after 24 h of growth suggests that some of the pyruvate might be channeled to ethanol production through oxidation to acetyl-CoA and subsequent reduction to ethanol, which would lead eventually to the re-oxidation of one molecule of NAD(P)H. However, if this is the case, the reason for using the last scenario instead of one-step reduction of pyruvate to lactate would be puzzling. The upregulation of the *pdh* operon in the presence of glucose instead of ribose was also observed in *L. gelidum* subsp*. gasicomitatum* [59], while in *L. sakei* [58] and *L. plantarum* [63] *pdh* operon had higher expression on ribose.

The upstream regions of all putative NAD(P)H re-oxidizing enzymes (except for lactate dehydrogenases), as well as some other glucose-induced genes, including glycerol dehydrogenase *gldA* and two glucose catabolism-related genes, were predicted to contain a motif similar to the binding site of a Rex transcriptional repressor. This regulator was found to negatively control the expression of genes involved in anaerobic/aerobic respiration, fermentation, oxidative stress response and central carbohydrate metabolism in several model Gram-positive bacteria [64–68] in response to NAD(P)+/NAD(P)H levels. Although being experimentally confirmed only in few species, Rex regulons were predicted to be widespread across six phyla of Gram-positive bacteria, including Firmicutes, where predicted Rex-regulated genes included mainly those involved in fermentation [49]. Rex repressor dissociates from DNA in response to decreased NAD(P)+/NAD(P)H ratio (can be achieved, for example, when glucose is fermented instead of pentoses), which leads to derepression, and, hence, upregulation of Rex-regulated genes involved mainly in NAD(P)H reoxidation through different pathways to restore redox balance [64–66]. The described mechanism agrees with the observations obtained for the *L. oligofermentans* genes associated with the Rex TFBS-like motif. Moreover, six such genes (*adhE*, *adhA* [Zn-dependent]*, nox*, *butA2*, *dhaT* and *gldA*) were also predicted to be part of Rex regulons in other *Lactobacillaceae* [49]. Therefore, our findings indirectly confirm the Rex-dependent regulation of redox balance in *Lactobacillaceae* predicted earlier [49], through the discovery of Rex TFBS-like sites in the upstream regions of strongly upregulated NAD(P)H re-oxidizing genes under low NAD(P)+/NAD(P)H ratio conditions (glucose versus pentoses). Although the genes involved in carbohydrate catabolism (however, not the same as in this study), as well as glycerol dehydrogenase *gldA* were also predicted to be part of Rex-regulons in other Firmicutes, their role in maintaining NAD(P)+/NAD(P)H levels remains unclear.

Pentose catabolism, in turn, proceeds without additional oxidation, which lessens the necessity of NAD(P)H re-oxidation. As a result, a pyruvate and acetyl-phosphate surplus is created, which leads to increased production of acetoin/diacetyl and acetate [44]. This explains the upregulation of the pathways involved in the production of acetoin/diacetyl and acetate observed in this study, when the growth medium contains pentoses instead of glucose. The same was also shown for *L. sakei* [58].

## Conclusions

With the aid of complete genome sequence and time course analysis of transcriptomes, this study provides insights into the transcriptional regulation of carbohydrate catabolism and heterolactic fermentation in the meat-borne obligate heterofermentative LAB *L. oligofermentans* that probably underlies its flexibility in terms of efficient use of carbohydrates available in the environment. The latter, together with other factors, identified in this study, could possibly be an important mechanism for the adaption of this bacterium to growth in meat, while, supposedly, being originally adjusted to a plant environment. The comparison of transcriptomes, obtained during the growth on three different carbohydrates, reveals intriguing metabolic features in this bacterium, such as synergistic upregulation of the same pathways by glucose and xylose, the absence of stringent CCC and potential involvement of CcpA regulator in it. Nevertheless, the exact mechanism of CcpA-related regulation requires further study. The obtained transcriptome response of an obligate heterofermentative *L. oligofermentans* to the growth on ribose instead of glucose differs significantly from that in another meat inhabiting but facultative heterofermentative bacterium *L. sakei*. This suggests a substantial difference in the regulation of carbohydrate catabolism and fermentation in these two groups of bacteria. Finally, the cell redox balance maintenance, in terms of NAD(P)+/NAD(P)H ratio, was predicted to be regulated by the Rex transcriptional regulator, supporting the previously made inference of Rex-regulons for the members of *Lactobacillaceae* family.

## Abbreviations

CCC, carbon catabolite control; CCR, carbon catabolite repression; CDS, coding DNA sequence; DE, differentially expressed; DHA, dihydroxyacetone; FC, fold change; HMM, hidden Markov model; LAB, lactic acid bacterium/bacteria; MAP, modified atmosphere packaged; MRS, de Man-Rogosa-Sharpe; OD_600_, optical density at 600 nm; PC(A), principle component (analysis); RPKM, reads per kilobase of gene per million mapped reads; TFBS, transcription factor binding site

## Additional files

Additional file 1: Table S1. Numbers of CDS- mapped reads per samples, used for the analysis. **Table S2.** The list of predicted putative adhesins in *L. oligofermentans* genome. **Table S3.** Predicted carbohydrate/carbon source catabolic/transport genes in *L. oligofermentans* genome. **Table S4.** Criteria for classifying genes into groups with different expression patterns. **Table S5.** The number of differentially expressed genes containing CcpA- binding site in the upstream regions for pairwise and triplewise comparisons between growth conditions. **Table S6.** Motif sequence LOGOs, discovered and enriched in the upstream regions of the co-regulated genes. **Figure S1.** Genome map of *L. oligofermentans* LMG 22743^T^. **Figure S2.** Venn diagrams representing the numbers of differentially expressed genes for pairwise and triplewise comparisons between growth conditions at 20 h (A), 24 h (B) and 30 h (C). **Figure S3.** RPKM plots for the putative glucose (*manXYZ*), xylose (*XylT*) and ribose (*deoP* and *rbsACB*) transporters. **Figure S4.** Aerobic growth on glucose, ribose and xylose, supplemented with heme or menaquinone or both. (PDF 953 kb) Additional file 2: Processed RNA-seq data and the results of differential expression analysis for three time points: 20 h (excel sheet 1), 24 h (excel sheet 2), 30 h (excel sheet 3). (XLSX 915 kb) Additional file 3: List of the genes containing CcpA-, RbsR- or XylR- binding motifs in their upstream regions. (XLSX 52 kb)
