# Supplementary material for: Lactobacillus oligofermentans glucose, ribose and xylose transcriptomes show higher similarity between glucose and xylose catabolism-induced responses in the early exponential growth phase
Source: BMC Genomics. 2016 Aug 3;17:539. doi: 10.1186/s12864-016-2840-x (PMC4972977; doi:10.1186/s12864-016-2840-x)
Supplement: Additional file 1: Table S1. — Numbers of CDS- mapped reads per samples, used for the analysis. Table S2. The list of predicted putative adhesins in L. oligofermentans genome. Table S3. Predicted carbohydrate/carbon source catabolic/transport genes in L. oligofermentans genome. Table S4. Criteria for classifying genes into groups with different expression patterns. Table S5. The number of differentially expressed genes containing CcpA- binding site in the upstream regions for pairwise and triplewise comparisons between growth conditions. Table S6. Motif sequence LOGOs, discovered and enriched in the upstream regions of the co-regulated genes. Figure S1. Genome map of L. oligofermentans LMG 22743T. Figure S2. Venn diagrams representing the numbers of differentially expressed genes for pairwise and triplewise comparisons between growth conditions at 20 h (A), 24 h (B) and 30 h (C). Figure S3. RPKM plots for the putative glucose (manXYZ), xylose (XylT) and ribose (deoP and rbsACB) transporters. Figure S4. Aerobic growth on glucose, ribose and xylose, supplemented with heme or menaquinone or both. (PDF 953 kb) [file 12864_2016_2840_MOESM1_ESM.pdf]

Additional file 1

Table S1. Numbers of CDS mapped reads per samples, used for the analysis.

	20 h	24 h	30 h
**Glucose**	7,283,742<br>1,344,898	1,654,086<br>759,538	3,158,152<br>818,617<br>842,725
**Ribose**	1,358,456<br>830,669<br>975,632	1,977,949<br>972,987<br>3,695,679	1,140,045<br>1,864,078
**Xylose**	13,839,497<br>1,637,634	1,799,644<br>1,107,260<br>2,202,281	1,883,860<br>804,435<br>873,722

Table S2. The list of predicted putative adhesins in *L. oligofermentans* LMG 22743^T genome.

Name of a putative adhesin	Locus_tag
**LPxTG-like motif-containing proteins**	
Mucus-binding protein	LACOL_1683
Mucus-binding protein	LACOL_1776
Collagen-binding protein	LACOL_1545
Cna_B domain-containing protein	LACOL_0719
Cna_B domain-containing protein	LACOL_1113
LPxTG-like motif-containing protein	LACOL_0133
LPxTG-like motif-containing protein	LACOL_0920
CscD-like protein	LACOL_0179
**Other putative adhesins**	
Fibronectin-binding protein	LACOL_0917
Ser-Asp repeat-containing proteins	LACOL_1668-1674
Ser-rich protein	LACOL_1675
Ser-rich protein	LACOL_0450
Ser-rich protein	LACOL_0277
Mucus-binding protein	LACOL_1109
Collagen triple helix repeat-containing protein	LACOL_0832
Collagen triple helix repeat-containing protein	LACOL_0833

Table S3. Predicted carbohydrate/carbon source catabolic/transport genes in *L. oligofermentans* LMG 22743^T genome.

**Carbon source**	**Predicted catabolic/transport genes**
D-Glucose	Putative glucose uptake permease (LACOL_1336)<br>PTS mannose-specific transporter (LACOL_0353-0355) [a]<br>Glucokinase (LACOL_0779)
D-Fructose	Fructokinase (LACOL_0264)<br>Glucose-6-phosphate isomerase (LACOL_1335)
D-Mannose	PTS mannose-specific transporter (LACOL_0353-0355)<br>Mannose-6-phosphate isomerase (LACOL_0639)
D-Galactose	Catabolic operon *galKTR* (LACOL_0218-0220)
D-Gluconate	Gluconate permeases (LACOL_1326, LACOL_1350)<br>Gluconokinase (LACOL_0566)
N-Acetyl-D-Glucosamin	PTS N-acetylglucosamine-specific transporter (LACOL_0524)<br>N-acetylglucosamine-6-phosphate deacetylase (LACOL_1463)<br>Glucosamine-6-phosphate deaminase (LACOL_1349)
D-Xylose	Putative xylose-proton symporters (LACOL_1587<br>LACOL_0262, LACOL_0266)<br>Catabolic operon *xylRAB* (LACOL_0400-0402)
D-Ribose	Ribose ABC transporter (LACOL_0445-0447)<br>(deoxy)Ribose permease (LACOL_0616)<br>Catabolic genes (LACOL_0442-0444; LACOL_0617-0619)
L-Arabinose	L-arabinose isomerase (LACOL_0275)<br>L-ribulokinase (LACOL_0256)
2-Deoxy-D-Ribose	(deoxy)Ribose permease (LACOL_0616)<br>(deoxy)Ribokinase (LACOL_0618)<br>Deoxyribose-phosphate aldolase (LACOL_1576)
Maltose	Transport/catabolic operon (LACOL_0921-0923)
Lactose	Beta galactosidases (LACOL_0521-0522; LACOL_1707)
L-ascorbate	Gene cluster for catabolism/transport (LACOL_0114-0122)
N-acetylneuraminate	Gene cluster for catabolism/transport (LACOL_1002-1007)
1,4-beta xylan derived oligosaccharides	Xyloside transporter (LACOL_0173)<br>Xylan 1,4-beta-xylosidase (LACOL_0172, LACOL_0427, LACOL_0431)
L-arabinan derived oligosaccharides	Arabinosidases (LACOL_0271, LACOL_0431, LACOL_ 1014)
Pyruvate	Pyruvate dehydrogenase (LACOL_1150-1153)
Glycerol	Glycerol transporters (LACOL_1374, LACOL_1480, LACOL_0234)<br>Glycerol dehydrogenase (LACOL_0123)<br>Putative DHA kinase (LACOL_0875)<br>Glycerol kinases (LACOL_0235, LACOL_0892)

	Putative glycerol-3-phosphate dehydrogenase (LACOL_1392)
Dihydroxy-acetone (DHA)	Putative DHA kinase (LACOL_0875)
Inosine, Adenosine, Uridine	Purine and pyrimidine nucleoside transporters (LACOL_1625, LACOL_1019)<br>Nucleoside phosphorylases (LACOL_1572, LACOL_1574)<br>Nucleoside hydrolases (LACOL_1017, LACOL_1624, LACOL_0326)<br>Phosphopentomutase (LACOL_1575)<br>Deoxyribose-phosphate aldolase (LACOL_1576)

[a] PTS mannose-specific transporter *manXYZ* (LACOL_0353-0355) might be involved in the transport of other hexoses, such as glucose and fructose [1]

Table S4. Criteria for classifying genes into groups with different expression patterns.

	**G/R**	**G/X**	**X/R**
**X ≠ G≈R**	-	DE	DE
**R ≠ G≈X**	DE	-	DE
**G ≠ R≈X**	DE	DE	-
**G ≠ R ≠ X**	DE	DE	DE

Designations used: G – glucose; R – ribose; X – xylose; G/R – the comparison between G and R transcriptomes; X ≠ G≈R – the group of genes that have distinct expression in xylose transcriptome in comparison with glucose and ribose transcriptomes, where the last two have relatively similar expression; G ≠ R ≠ X – the group of genes that have distinct expression levels in all three transcriptomes; DE – statistically significant differential expression (adjusted *p-value* =< 0.05 and absolute log2 FC=>1).

Table S5. The number of differentially expressed genes containing CcpA-binding sites in their upstream regions for the pairwise and triplewise comparisons between growth conditions.

	No. of genes, total	Up	Down
**Pairwise comparisons**[a]			
G/R	37	30 (81%)	7 (19%)
G/X	10	7 (70%)	3 (30%)
X/R	45	34 (75%)	11 (25%)
**Triplewise comparisons**[b]			
X ≠ G≈R	1	1	0
R ≠ G≈X	25	6 (25%)	21 (75%)
G ≠ R≈X	4	4	0
G ≠ R ≠ X	3[c]		

[a] G indicates glucose; R – ribose; X – xylose. Notation G/R means the comparison between glucose and ribose transcriptomes, where up- and downregulation is given for glucose transcriptome relative to ribose transcriptome;

[b] X ≠ G≈R designates the group of genes that have distinct expression in xylose transcriptome in comparison with glucose and ribose transcriptomes, where the last two have relatively similar expression; up- and downregulation is given for xylose transcriptome relative to glucose and ribose transcriptome; G ≠ R ≠ X – the group of genes that have distinct expression levels in all three transcriptomes.

[c] in all three G ≠ R ≠ X cases the expression of the genes were higher on glucose and xylose in comparison with ribose: X>G>R (2) and G>X>R (1).

Other designations used: Up – upregulation, Down – downregulation.

Table S6. Motif sequence LOGOs, discovered and enriched in the upstream regions of the co-expressed genes.

**Groups of co-expressed genes**[a]	**Motif sequence LOGOs**	**Enrichment *p-value***	**TFBS database match (*q-value*[b])**
G > R, 20 h	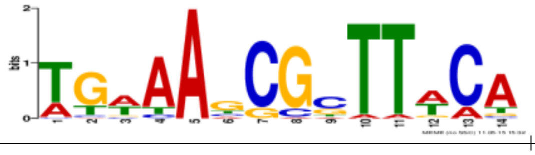	6.58e-6	*Lactobacillaceae* CcpA (1e-07)<br>*Bacilli* CcpA (1e-06)
X > R, 20 h	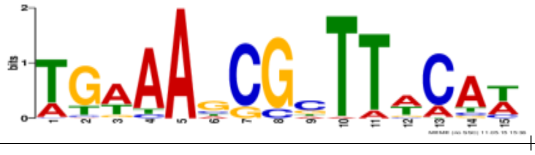	1.76e-10	*Lactobacillaceae* CcpA (2e-08)<br>*Bacilli* CcpA (4e-07)
G ≈ X > R, 20 h	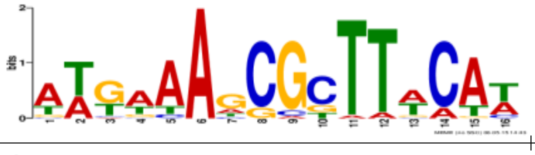	1.04e-5	*Lactobacillaceae* CcpA (1e-09)<br>*Bacilli* CcpA (2e-07)
G > X, 20 h	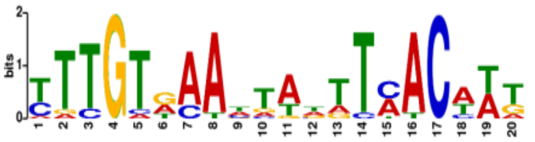	1.32e-5	*Bacillales* Rex (9e-08)<br>*Lactobacillaceae* Rex (1e-07)<br>*Bacillus* Fnr (2e-03)
G > X, 24 h	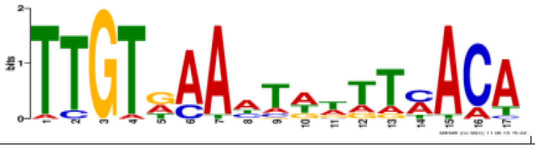	1.92e-5	*Bacillales* Rex (2e-09)<br>*Lactobacillaceae* Rex (7e-09)<br>*Bacillus* Fnr (6e-05)

[a] G indicates glucose; R – ribose; X – xylose; Notation G > R designates the group of genes that have higher expression during the growth on glucose tan on ribose; G ≈ X > R – the group of genes that have similar expression levels on glucose and xylose, which are higher than on ribose.

[b] *q-value* is an adjusted *p-value*, as given by *tomtom* tool from MEME suite [2]

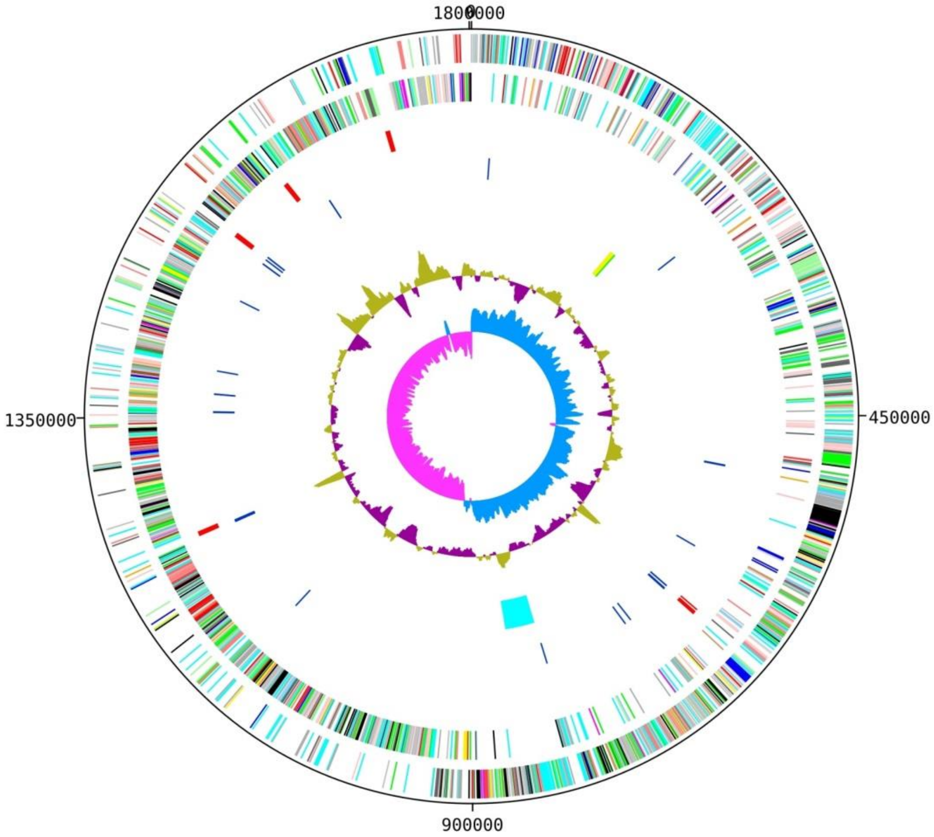

Chromosome size (bp)	1,801,673
GC content (%)	35.6
CDSs	1722
Unknown function (%)	15
rRNA operons	5 operons
tRNA genes	55
tmRNA genes	1
Prophages (complete)	1
CRISPR/Cas system	type IIA

Figure S1. Genome map of *L. oligofermentans* LMG 22743^{T}. Genes are colored accordingly with their COG functional annotation. Moving inwards, the tracks represent the following features: genes on the forward strand, genes on the reverse strand, rRNA genes (red), tRNA genes (blue), prophage (cyan) and CRISPR/Cas system (yellow and green), GC-plot (purple and yellow), and GC-skew (pink and blue).

A. Time: 20 h

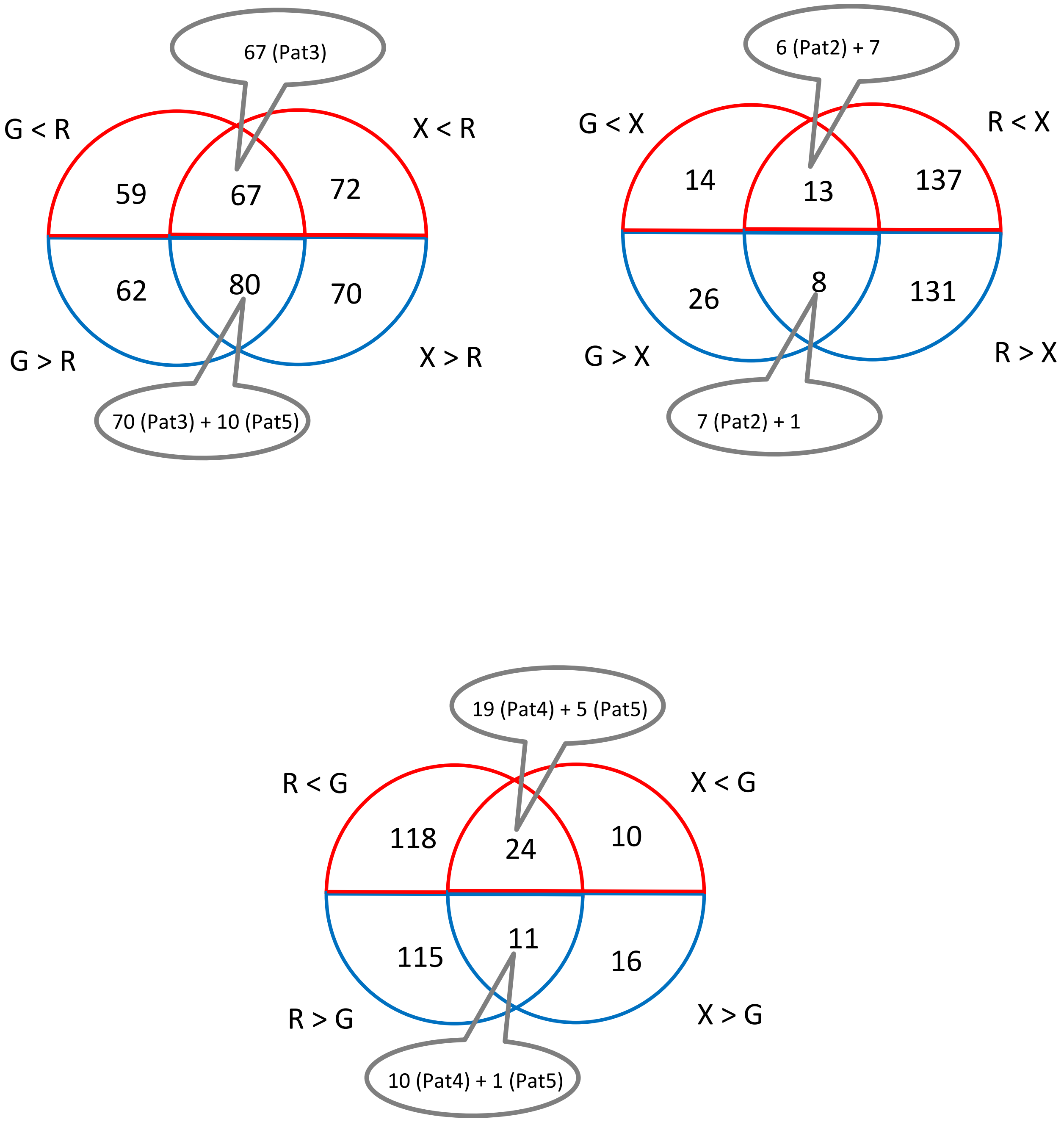
67 (Pat3)
G < R
X < R
59
67
72
62
80
70
G > R
X > R
70 (Pat3) + 10 (Pat5)
6 (Pat2) + 7
G < X
R < X
14
13
137
26
8
131
G > X
R > X
7 (Pat2) + 1
19 (Pat4) + 5 (Pat5)
R < G
X < G
118
24
10
115
11
16
R > G
X > G
10 (Pat4) + 1 (Pat5)

B. Time: 24 h

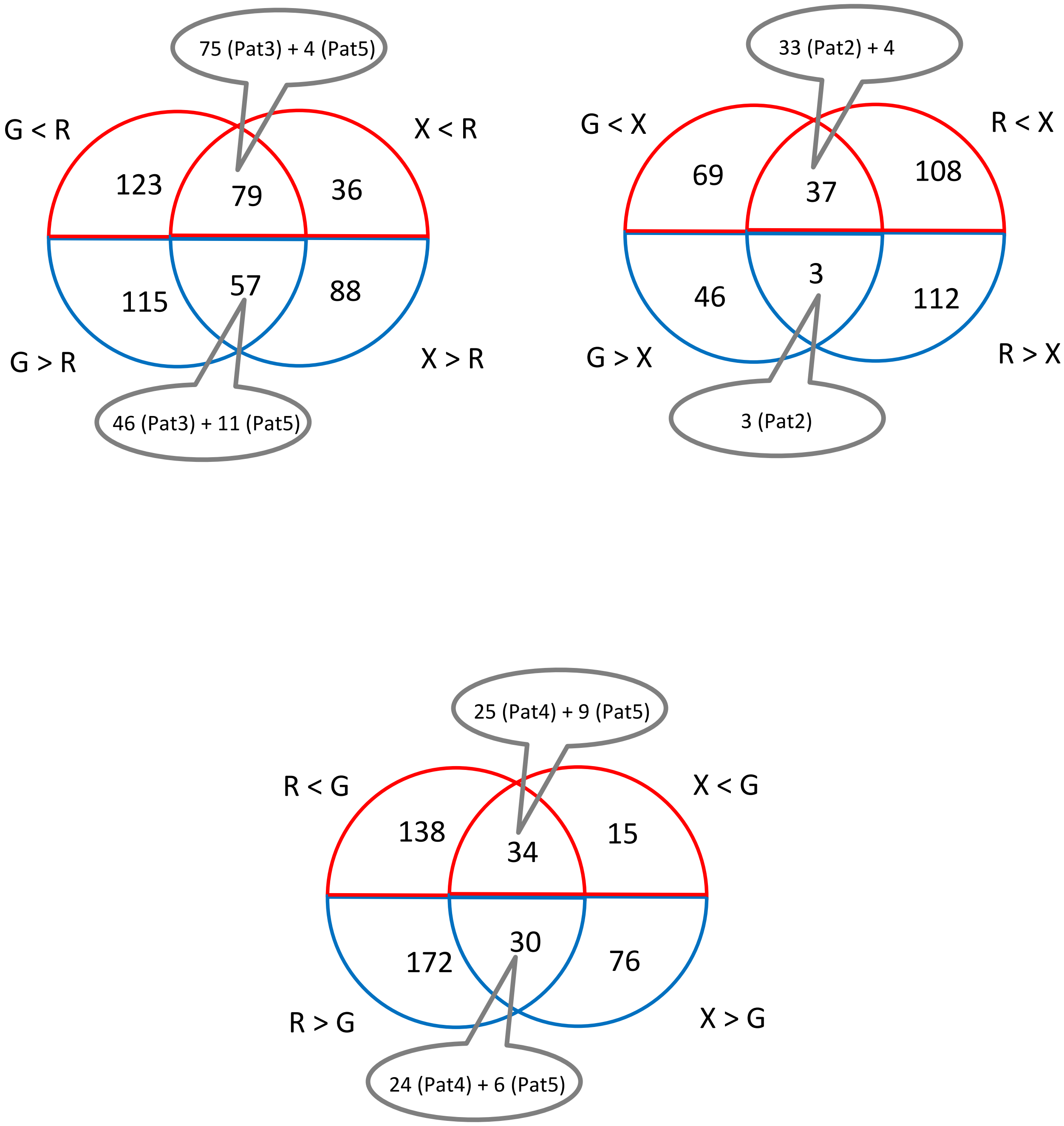
75 (Pat3) + 4 (Pat5)
G < R
X < R
123
79
36
115
57
88
G > R
X > R
46 (Pat3) + 11 (Pat5)
33 (Pat2) + 4
G < X
R < X
69
37
108
46
3
112
G > X
R > X
3 (Pat2)
25 (Pat4) + 9 (Pat5)
R < G
X < G
138
34
15
172
30
76
R > G
X > G
24 (Pat4) + 6 (Pat5)

C. Time: 30 h

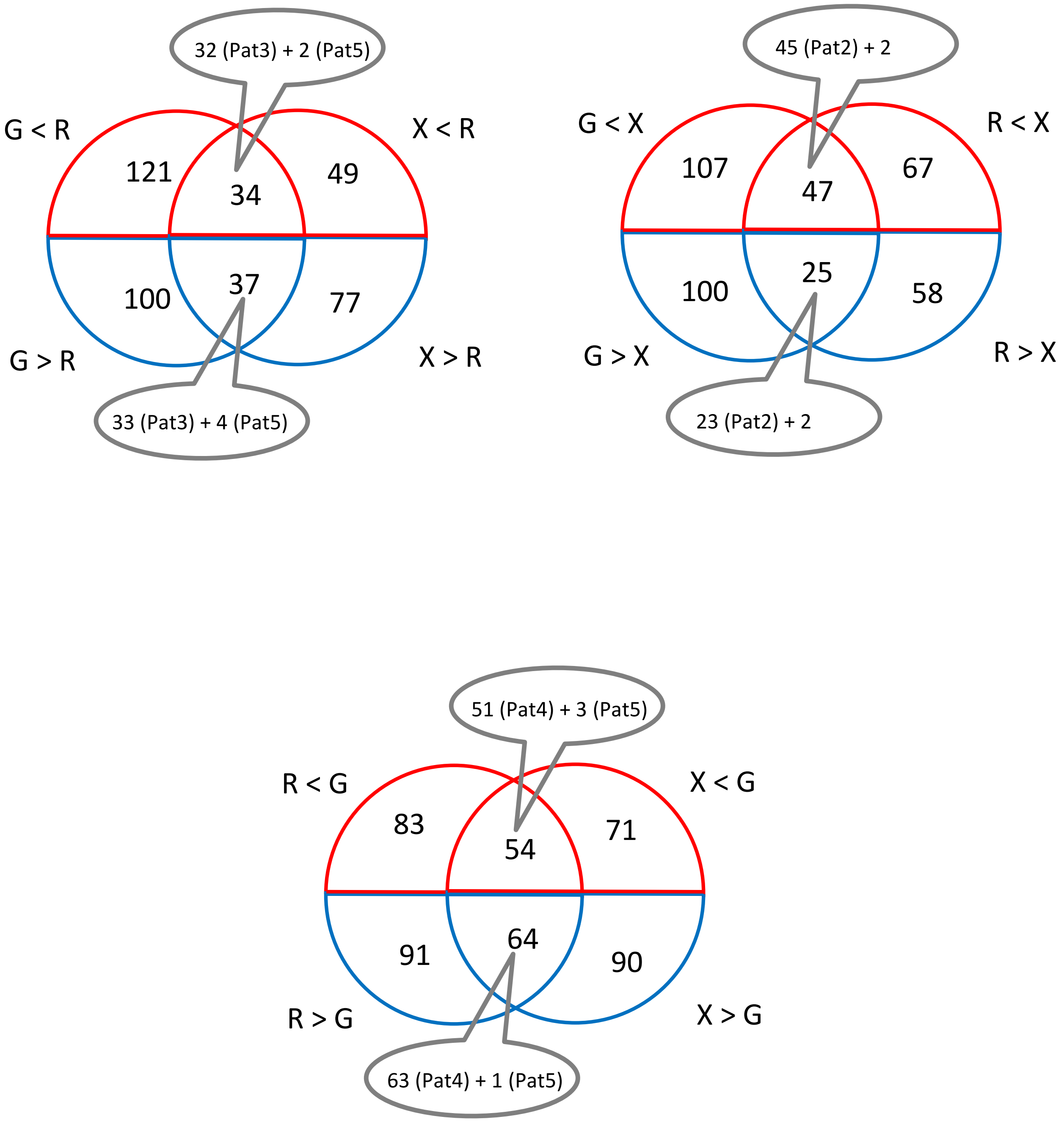

Figure S2. Venn diagrams representing the numbers of differentially expressed genes for pairwise and triplewise comparisons between growth conditions at 20 h (A), 24 h (B) and 30 h (C). G indicates glucose; R – ribose; X – xylose. Notation G > R means statistically significant higher expression in G in comparison with R. Expression patterns: Pat1: $X \approx G \approx R$; Pat2: $X \neq G \approx R$; Pat3: $R \neq G \approx X$; Pat4: $G \neq R \approx X$; Pat5: $G \neq R \neq X$. For more detailed information about patterns and criteria, used to classify genes into them, can be found in Additional file 1: Table S4.

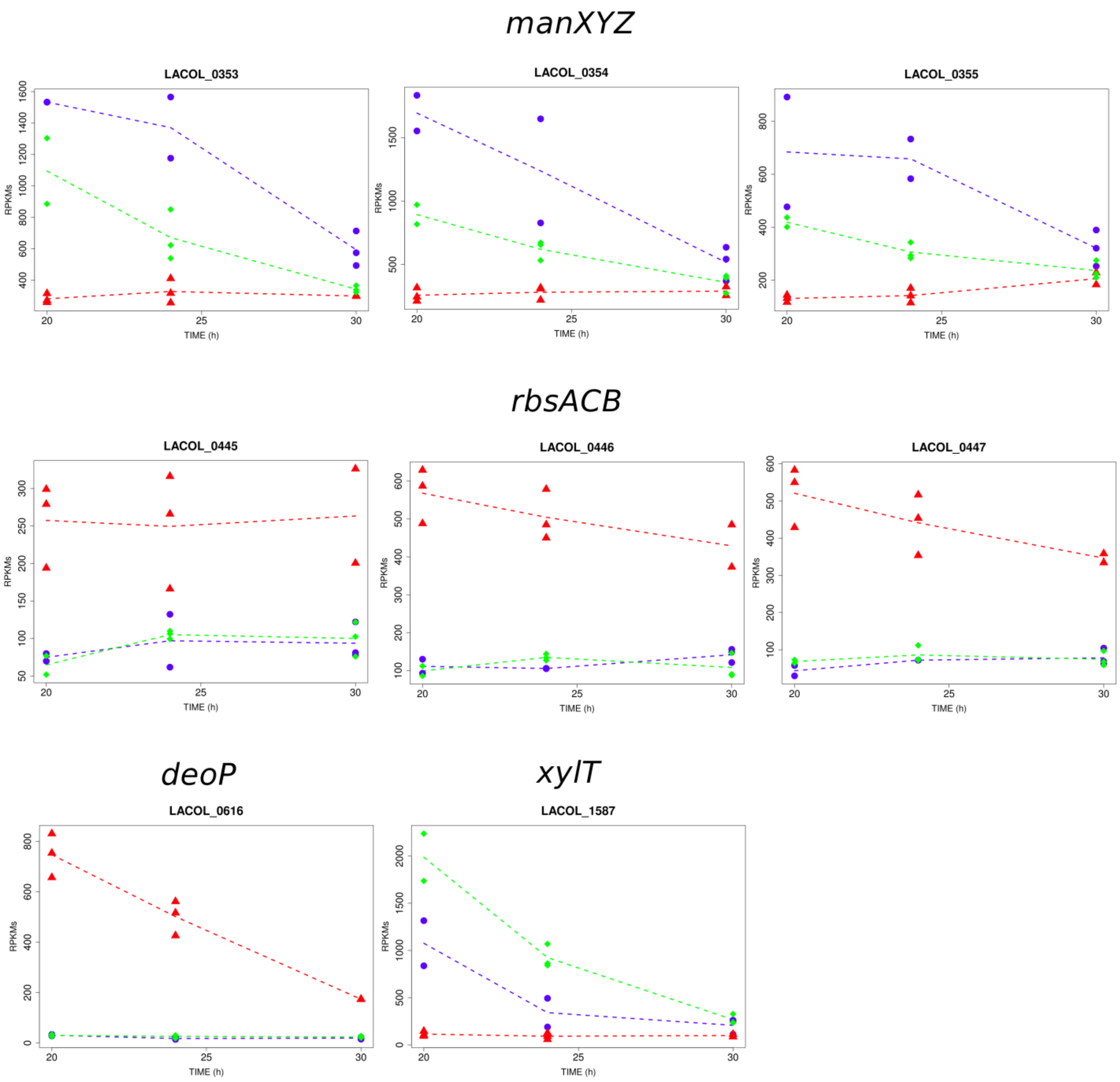

Figure S3. RPKM plots for the putative glucose (*manXYZ*), xylose (*XylT*) and ribose (*deoP* and *rbsACB*) transporters. Each point represents an RPKM value for one sample, obtained during the growth on glucose (blue dots), xylose (green diamond), ribose (red triangle). Dashed lines connect the average values across samples obtained during the growth on a certain carbohydrate for each time point.

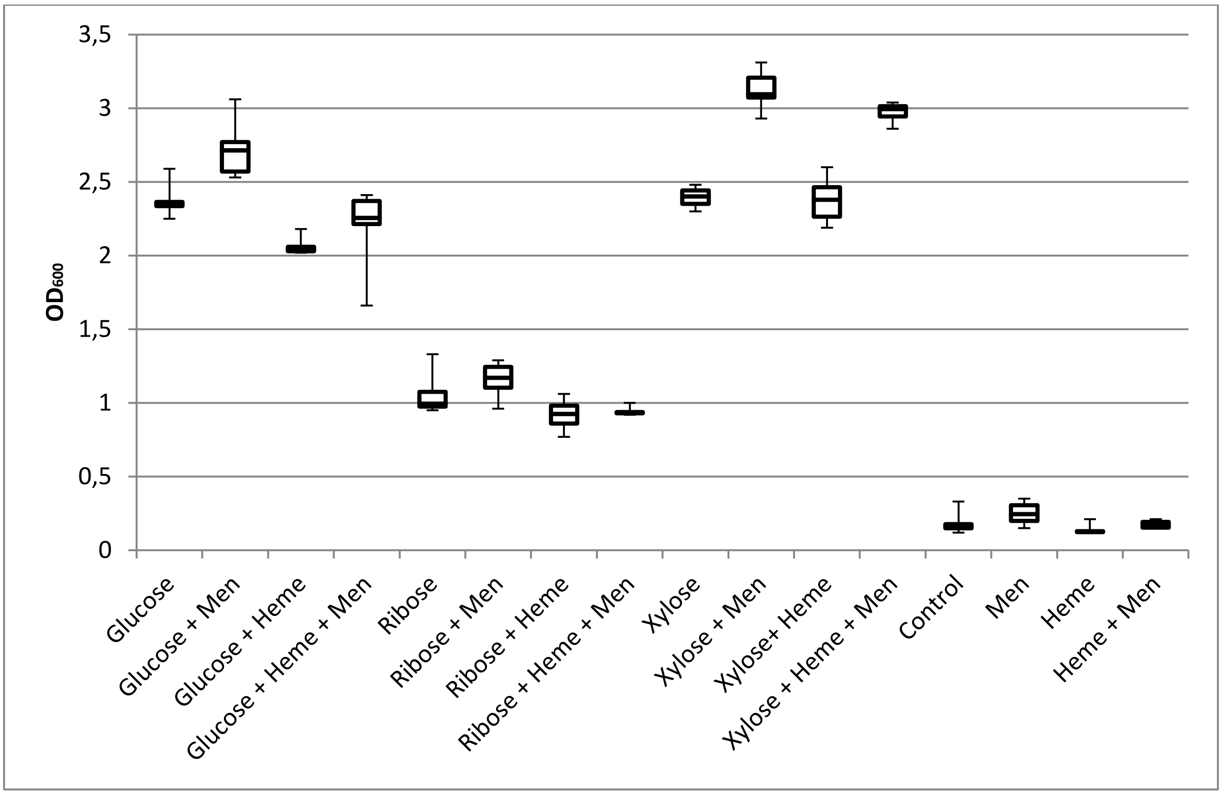

Figure S4. Aerobic growth on glucose, ribose and xylose, supplemented with heme or menaquinone or both. *L. oligofermentans* LMG 22743^{T} was grown in six replicate per each growth condition and data are represented as box-and-whisker plot. Designations used: Men – menaquinone, Control – growth without carbohydrate.

.

References:

1. Lauret R, Morel-Deville F, Berthier F, Champomier-Verges M, Postma P, Ehrlich S, Zagorec M: **Carbohydrate Utilization in *Lactobacillus sake***. *Appl Envir Microbiol* 1996, **62**:1922–1927.

2. Bailey TL, Boden M, Buske FA, Frith M, Grant CE, Clementi L, Ren J, Li WW, Noble WS: **MEME SUITE: tools for motif discovery and searching.** *Nucleic Acids Res* 2009, **37**(Web Server issue):W202–8.
